# Acute Effects of an Anthocyanin-Rich Blackcurrant Beverage on Markers of Cardiovascular Disease Risk in Healthy Adults: A Randomized, Double-Blind, Placebo-Controlled, Crossover Trial

**DOI:** 10.1016/j.tjnut.2025.05.017

**Published:** 2025-05-23

**Authors:** Anna M Amini, Ruihan Zhou, Katharina Austermann, Dominika Králová, Gessica Serra, Ibrahim S Ibrahim, Giulia Corona, Triana Bergillos-Meca, Hassan Aboufarrag, Paul A Kroon, Jeremy PE Spencer, Parveen Yaqoob

**Affiliations:** 1 Department of Food and Nutritional Sciences, Hugh Sinclair Unit of Human Nutrition, University of Reading, Reading, United Kingdom; 2 School of Life and Health Sciences, Whitelands College, University of Roehampton, London, United Kingdom; 3 Quadram Institute Bioscience, Norwich Research Park, Norwich, United Kingdom

**Keywords:** anthocyanins, blackcurrant, blood pressure, inflammation, vascular function

## Abstract

**Background:**

Epidemiologic evidence suggests an inverse association between anthocyanin consumption and cardiovascular disease (CVD) risk. Modulation of vascular function and hemostasis may contribute to this, but there is limited clinical evidence.

**Objectives:**

The present study investigated the acute effects of an anthocyanin-rich blackcurrant beverage, compared with a matched placebo, on selected markers of CVD risk in healthy middle-aged subjects in response to a high-fat meal.

**Methods:**

Twenty-three volunteers aged 39.9 ± 8.1 y [body mass index (BMI) (in kg/m^2^) 22.9 ± 2.3] completed a double-blind, randomized, placebo-controlled, crossover trial. Volunteers consumed either a 200 mL blackcurrant beverage (744 mg polyphenols comprising 711 mg anthocyanins and 32 mg procyanidins) or a placebo, together with a high-fat breakfast (52.3 g fat) followed by a lunch (30 g fat) at 3 h, and the postprandial vascular response was compared. The primary endpoints were the assessment of vascular function by flow-mediated dilation (FMD) and the inhibition of collagen- and adenosine diphosphate-induced platelet aggregation. Secondary outcomes included blood pressure (BP), digital volume pulse waveforms, circulating numbers of endothelium- and platelet-derived extracellular vesicles, plasma concentrations of interleukin (IL)-8, and plasma and urinary concentrations of polyphenols and their metabolites were also evaluated.

**Results:**

There was a significant cumulative improvement in FMD following consumption of an anthocyanin-rich blackcurrant beverage compared with a matched placebo in conjunction with a high-fat meal over a 6 h postprandial period. There was a trend for an inhibitory effect of the blackcurrant beverage on agonist-induced platelet aggregation and significant effects on the secondary outcomes, systolic BP and IL-8, although these were exploratory and not adjusted for multiple testing. Plasma concentrations of hippuric acid and isovanillic acid were strong independent predictors of FMD, and 4-hydroxybenzaldehyde and isoferulic acid glucuronide were predictors of systolic BP and diastolic BP.

**Conclusions:**

An anthocyanin-rich blackcurrant beverage mitigates the effects of a high-fat meal on vascular function and markers of CVD risk, and this is associated with the appearance of specific plasma anthocyanin phenolic metabolites.

This trial was registered at classic.clinicaltrials.gov as NCT02459756.

## Introduction

Anthocyanins are abundant in berry fruits and belong to the flavonoid group of polyphenols. A cardioprotective benefit of flavonoid-rich foods has been demonstrated in several studies [1]. However, the most convincing clinical evidence exists only for a few flavonoid-rich products (chocolate, black, and green tea), which have been extensively studied. Consumption of these flavonoid sources has been reported to exert beneficial effects on some risk factors for cardiovascular disease (CVD), such as LDL cholesterol and blood pressure (BP), as well as on vascular function and platelet aggregation. Epidemiologic evidence has also linked consumption of anthocyanins with a lower risk of CVD [[2], [3], [4]] and CVD risk markers [[5], [6], [7]], but only a few clinical intervention trials have investigated the effect of anthocyanin-rich interventions on CVD risk markers [8]. Blueberry polyphenols have been demonstrated to improve vascular function [flow-mediated dilation (FMD)] in healthy males, and the biphasic increase in FMD observed at 1–2 and 6 h posttreatment was paralleled by the appearance of phenolic compounds in plasma and inhibition of neutrophil Nicotinamide adenine dinucleotide phosphate (NADPH) oxidase activity [9]. However, the blueberry intervention delivered other polyphenol classes (predominantly procyanidins and chlorogenic acid) in addition to anthocyanins, and the nature of their bioactivity is unclear.

There are several further potential mechanisms by which anthocyanins and other phytochemicals may reduce the risk of CVD, including modulation of platelet activation and aggregation, number of circulating extracellular vesicles (EVs), cytokine production, and arterial stiffness. There is consensus on the predictive value of platelet activity on CVD risk [[10], [11], [12]], and a recent review of human randomized controlled trials (RCTs) concludes that there is strong evidence for an antiplatelet effect of flavan-3-ol-rich cocoa (products) and grape seed extract, particularly after acute consumption [13], but little is known about the effect of anthocyanin-rich interventions.

Elevated numbers of circulating EVs, which are small plasma membrane vesicles shed from the surface of a variety of stimulated and/or apoptotic cells, are suggested to indicate vascular injury and inflammation as an emerging risk marker for CVD [14,15]. Consumption of cocoa polyphenols reduces the number of circulating endothelial-derived EVs (EDEVs) in patients with coronary artery disease [16], as well as females with overweight/obesity, although no effects were observed in females with normal weight [17]. To our knowledge, no RCTs have investigated the effect of anthocyanins on the number of circulating EVs. There is also little information regarding the effects of anthocyanins following a vascular challenge, such as a high-fat meal. The aim of the present study was to compare in a healthy, middle-aged population the effects of an anthocyanin-rich blackcurrant beverage with a control beverage on a range of CVD risk markers following a high-fat meal challenge.

## Methods

The study was recruited and conducted at the Hugh Sinclair Unit of Human Nutrition at the University of Reading from June to November 2015. Written informed consent was obtained from all subjects. The study was conducted according to the guidelines laid down in the Declaration of Helsinki, approved by the University of Reading Research ethics committee (reference: UREC 14/17) as well as GlaxoSmithKline and registered at clinicaltrials.gov as NCT02459756.

### Participants

**FIGURE 1:**
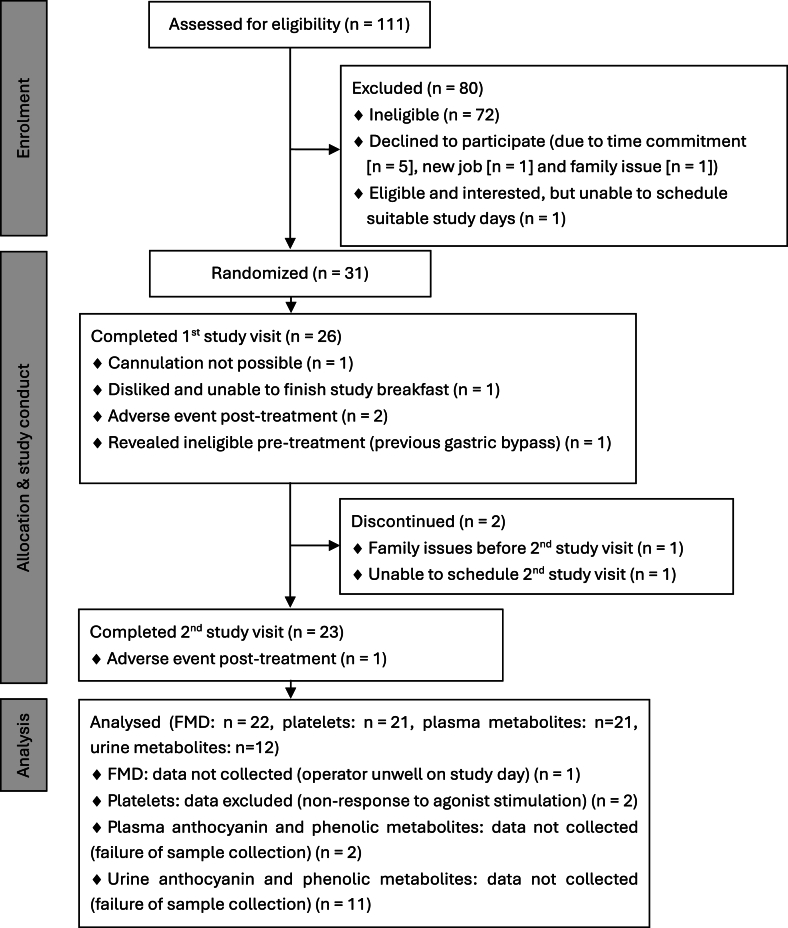
Participant flow. FMD, flow-mediated dilation.

Volunteers were recruited from Reading (United Kingdom) and surrounding areas through email, posters, and internet advertisements; the participant flow diagram is depicted in Figure 1. A total of 23 volunteers completed the study (39.87 ± 8.13 y old; 11 females). Their baseline characteristics are summarized in Table 1. Eligible for participation were healthy, nonsmoking males and females aged 30–55 y, with a normal to overweight BMI (20–30 kg/m^2^). Exclusion criteria included systolic BP (SBP) >140 mm Hg; diastolic BP (DBP) >90 mm Hg; total cholesterol >6.2 mmol/L; anemia (hemoglobin concentrations <130 g/L in males and <120 g/L in females); presence of diabetes mellitus, heart problems, stroke, vascular, inflammatory, kidney, liver, pancreas, or gastrointestinal diseases; usage of medication for hyperlipidemia, hypertension, hypercoagulation, or inflammatory conditions; usage of aspirin >2 times/wk; unable to abstain from aspirin ingestion for 14 d prior to each study visit; usage of antibiotics in the previous 3 mo before the study; participation in intense aerobic exercise (>20 min, 3 times a week); pregnancy or breastfeeding; usage of phytochemical, antioxidant or fish oil supplements, veganism, alcohol misuse or intakes >21 units/wk for males and >15 units/wk for females or participation in another clinical trial. Full blood count parameters and markers of liver and kidney function were confirmed to be within the normal range prior to entry into the study.

**TABLE 1:** Clinical characteristics of subjects who completed the study.

Baseline characteristic	Blackcurrant beverage first *n* = 12	Placebo beverage first *n* = 11
Age (y)	37 ± 7.3	43 ± 8.3
Male: female ratio	7:5	5:6
BMI (kg/m^2^)	22.7 ± 2.4	23.1 ± 2.0
Body fat (%)	21.0 ± 5.2	25.2 ± 6.0
SBP (mm Hg)	117.0 ± 12.1	119.5 ± 11.9
DBP (mm Hg)	71.8 ± 6.9	71.2 ± 9.6
Serum total cholesterol (mmol/L)	5.0 ± 1.0	4.9 ± 1.7
Serum fasting glucose (mmol/L)	5.2 ± 0.3	4.9 ± 0.3

Data are shown as mean ± SD.

Abbreviations: BMI, body mass index; DBP, diastolic blood pressure; SBP, systolic blood pressure; SD, standard deviation.

### Trial design

A randomized, double-blind, placebo-controlled crossover intervention trial was performed in which volunteers were asked to consume each of the following 2 treatments in random order in conjunction with a high-fat meal: blackcurrant beverage (200 mL containing 711 mg anthocyanins, Table 2 [18**]**) and a matched placebo beverage. Treatment order randomization was conducted by minimization using the stratification variables sex (male/female), age (30–42 y / 43–55 y), and BMI (20–24.9 and 25–30) based on the fact that subjects were healthy and had been comprehensively screened against the above exclusion criteria. The randomization code was generated using Excel, and randomization was concealed from both researchers and participants by having the drink powders independently labeled “A” and “B” before the start of the trial and using opaque cups with lids to conceal any minor differences between the appearance of the drinks. The study visits took place at the Hugh Sinclair Unit of Human Nutrition at the University of Reading, separated by 4 wk for females (to control for possible effects of the menstrual hormones on vascular function, but not standardized to a specific phase of the cycle) or ≥3 wk for males. Subjects were asked to refrain from exercise, caffeine, and alcohol and to follow a low-flavonoid diet for 24 h before the visit and throughout the study day. Subjects were provided with a low-fat, low-flavonoid meal for consumption on the evening before each study day. Subjects arrived at the clinical unit after an overnight fast (12 h), and a 24 h dietary recall was conducted to verify compliance with the low-flavonoid diet. A cannula was inserted into the forearm, and blood samples were collected at baseline and at 1 h, 2 h, 4 h, 6 h, and 24 h posttreatment. Polyphenol and metabolite analysis was performed at all timepoints; IL-8 analysis at baseline and at 1 h, 2 h, 4 h, and 6 h posttreatment; platelet aggregation and EV analysis at baseline and at 2 h and 4 h posttreatment. Vascular measurements [FMD, BP, and digital volume pulse (DVP)] were performed at baseline and at 2 h, 4 h, and 6 h posttreatment. FMD and BP were additionally measured at 1 h posttreatment. Urine was collected at baseline and at 0–1 h, 1–2 h, 2–4 h, 4–6 h, and 6–24 h posttreatment for polyphenol and metabolite analysis. The total volume of urine produced during each time period was recorded. After the baseline measurements were taken, subjects consumed, within 15 min, the test beverage together with a standardized, high-fat, low-flavonoid breakfast (756.1 kcals, 52.3 g fat of which 32.9 g was saturated, 57.9 g carbohydrates and 11.3 g protein) consisting of 2 croissants with butter. A standardized low-flavonoid lunch (665.5 kcals, 30.0 g fat of which 12 g was saturated, 81.1 g carbohydrates, and 15.1 g protein) consisting of a soft cheese sandwich, ready salted crisps, and 2 shortbread biscuits were provided at 3 h after the consumption of the test beverage and breakfast, and a low-fat, low-flavonoid meal was provided for dinner. Low-nitrate water (Buxton) was provided to subjects during the whole intervention period as needed. Researchers involved in the measurement and assessment of study outcomes generated the random allocation sequence, enrolled the participants, and assigned the interventions, but they were blinded to the allocation of treatment orders; all participants were blinded to the interventions as well.

**TABLE 2:** Composition of the test beverages (per serving).

Parameter	Blackcurrant beverage	Placebo beverage
**Polyphenols (mg)**		
Total polyphenols	744	0
Total anthocyanins	711	0
Delphinidin-3-O-rutinoside	349	0
Cyanidin-3-O-rutinoside	207	0
Delphinidin-3-O-glucoside	102	0
Cyanidin-3-O-glucoside	33	0
Petunidin-3-O-rutinoside	7.7	0
Petunidin-3-(6-coumaroyl)- glucoside	7.7	0
Peonidin-3-O-rutinoside	4.9	0
Total procyanidins	32	0
Monomers	6.9	0
Oligomers	26	0
**Macronutrients (g)**		
Sucrose	1.13	1.13
Glucose	0.62	0.62
Fructose	0.72	0.72
Maltodextrin	0.46	0.46
Protein	0.15	0
Fat	0.01	0
**Organic acids**		
Total (g)	0.50	0.50
Vitamin C (mg)	1.72	1.72
**Minerals (mg)**		
Potassium	34.73	0
Calcium	12.51	0
Magnesium	4.31	0
Sodium	1.03	0
Phosphorus	5.22	0
**Other**		
Artificial berry flavoring (g)	0.20	0.20
Low-nitrate water (Buxton) (mL)	200	200
Fiber (g)	0.07	0

Quantification of polyphenols was conducted by HPLC as described in Supplementary Methods, whereas BerryPharma provided information on other constituents in the blackcurrant powder. Except for vitamin C, there was no detailed information available on the type and proportion of organic acids in the blackcurrant powder; hence, available data on the organic acid content in blackcurrants from the literature were used [18] to formulate the placebo beverage (0.3% vitamin C, 6% malic acid and 94% citric acid).

Abbreviation: HPLC, high-pressure liquid chromatography.

### Test beverages

Table 2 details the composition of the test beverages. The intervention beverage contained 744 mg polyphenols, of which 711 mg were anthocyanins (49% delphinidin-3-O-rutinoside, 29% cyanidin-3-O-rutinoside, 14% delphinidin-3-O-glucoside, 5% cyanidin-3-O-glucoside, and 3% other anthocyanins) and 32 mg procyanidins and was comprised of spray-dried blackcurrant powder (*Ribes nigrum*; BerryPharma, product 70140015, batch L 14IV04342, stored in the dark at −20°C until use), sucrose (Tate & Lyle) and artificial blackcurrant flavoring (International Flavours & Fragrances) dissolved in 200 mL of low-nitrate water. The placebo beverage was devoid of polyphenols and was matched for sugars [sucrose, glucose, and fructose (all Myprotein)] and organic acids [citric acid (Sigma Aldrich)], vitamin C, and malic acid (both Myprotein). In addition, artificial blackcurrant flavoring was added to both beverages to mask taste differences. Study beverages were analyzed for anthocyanin and procyanidin content, the main polyphenol classes that have previously been reported in blackcurrants [19]. Quantifications of anthocyanins and procyanidins in the beverages were conducted by (HPLC)/mass spectrometry (MS), as described in Supplemental Methods, whereas information on other constituents in the blackcurrant powder was provided by BerryPharma. The powdered beverage ingredients were weighed out and packaged as individual servings at the University of Reading, coded A or B by an independent researcher, and stored in the dark at −20°C until use. Encoding was broken after all data analysis had been completed. On study days, the beverages were prepared by an independent researcher immediately before consumption by dissolving in 200 mL of low-nitrate water. Beverages were served in identical nontransparent lidded containers with a black straw to conceal color differences.

### Blood collection and processing

Blood samples were collected into K_3_EDTA tubes (for EVs and polyphenols and their metabolites), lithium heparin tubes (for IL-8), and sodium citrate tubes (for platelet aggregation). After blood sample collection, sodium citrate tubes were stored at room temperature, whereas the other tubes were kept on ice. Blood samples for the analysis of IL-8 and polyphenols and metabolites were centrifuged at 1700 × *g*; 15 min; 4°C. Samples were divided into aliquots and stored at −20°C (for IL-8 analysis) or −70°C (for polyphenol and metabolite analysis). Plasma for polyphenol and metabolite analysis was acidified with 2% formic acid (Sigma Aldrich) before storage to prevent anthocyanin degradation [9]. Urine was supplemented with 100 mg ascorbic acid (Sigma Aldrich) per 500 mL urine and acidified with formic acid to pH 2.4 before storage [20].

### Assessment of vascular function and BP

Measurements were performed in a quiet, temperature-controlled (24 ± 2°C) room. The subject lay quietly for several minutes before the measurements were performed.

Brachial artery FMD was measured according to established guidelines [21]. A Phillips CX50 integrated ultrasound system (Phillips) utilizing a 15–7 MHz transducer (L15-7io Broadband Compact Linear Array transducer; Phillips) was used in combination with a semi-automated analysis system (brachial analyzer; Medical Imaging Applications). Briefly, the brachial artery was imaged at 2–10 cm proximal to the antecubital fossa in the longitudinal plane. After recording baseline arterial diameter for 60 s, reactive hyperemia was induced by 5 min of lower arm occlusion through inflation of a cuff to 220 mm Hg. Data collection continued for 5 min after cuff release. A single researcher analyzed all image files. The peak diameter was defined as the mean of the 3 biggest diameters obtained after the occlusion was released. FMD was calculated as the percentage change in the brachial artery diameter from baseline to the peak diameter after cuff release. Each frame was analyzed in triplicate, and the mean was used in the statistical analysis. All FMD measurements were performed by the same trained operator.

DVP waveforms were measured in the semi-supine position using a PulseTrace PCA 2 device (CareFusion) by finger photoplethysmography. The device automatically analyzes DVP waveforms and calculates the stiffness index (DVP-SI) and the reflection index (DVP-RI). DVP-SI is a measure of large artery stiffness [22], whereas DVP-RI is a measure of the vascular tone of the small arteries [23].

BP was measured on the upper arm using an automated sphygmomanometer (Omron M6 Comfort; Omron Corporation) with the subject in a supine position after 5 min of rest. Three consecutive measurements were recorded, and the mean was used in the statistical analysis.

### Assessment of agonist-induced ex vivo platelet aggregation

Citrated blood for platelet function measurement was collected via a cannula at baseline as well as 2 h and 4 h posttreatment, and measurements were performed within 90 min of blood collection. Platelet-rich plasma (PRP) was prepared by centrifugation at 102 × *g* for 20 min. Following the removal of PRP, another centrifugation step was performed at 1413 × *g* for 10 min to provide platelet-poor plasma (PPP). Platelets in PRP were counted and standardized using a Z2 Coulter counter (Beckman Coulter). Platelet aggregation was induced in PRP (225 uL) with 2 agonists: collagen (0.5 and 1 *μ*g/mL final concentration; Takeda) and ADP (10 *μ*M and 100 *μ*M final concentration; Sigma Aldrich). Measurements were performed in triplicate in a chronolog optical platelet aggregometer (Chrono-log) with constant stirring at 1200 × *g* and at 37°C.

### Assessment of plasma cytokine concentration

Plasma concentrations of IL-8 were measured using an enhanced sensitivity cytometric bead array kit from BD Biosciences according to the manufacturer’s instructions. Data were acquired on a BD FACSCanto II flow cytometer (BD Biosciences) and analyzed using the BD FCAP Array v3 software. The limits of detection of these cytokine assays are 0.274 pg/mL. Analysis of IL-1β, TNF-α, IL-6, and IL-10 was discontinued following sub-analysis of 5 volunteers where most data were below the limit of detection.

### Assessment of EVs

Numbers of circulating EDEVs and platelet-derived EVs (PDEVs) were quantified as previously described [24]. In brief, PPP was stained with CD42b conjugated to fluorescein isothiocyanate and CD31 conjugated to phycoerythrin antibodies in TruCount tubes (all from BD Biosciences). After an incubation period, samples were diluted with phosphate-buffered saline, and data was acquired on a BD Accuri flow cytometer (BD Biosciences) using the gating strategy described in detail in Wu et al. [24]. EDEVs were defined as CD31+CD42b− and PDEVs as CD31+CD42b+. Values are expressed as counts per microliter of PPP.

### Anthocyanin and phenolic metabolites analysis in plasma and urine

Identification and quantification of anthocyanin and phenolic metabolites were conducted by ultra-performance liquid chromatography-MS/MS following solid phase extraction as described in Supplemental Methods and Supplemental Table 1.

### Power calculation

The sample size calculation was performed according to Julious et al. [25] for the primary endpoints of brachial artery FMD and agonist-induced platelet aggregation. For the former, 15 subjects would provide 80% power in detecting a 0.7% absolute difference in brachial artery FMD with α = 0.05 and a within-subject SD of 0.6. For platelet function, 21 subjects would provide 80% power in detecting a difference of 6% or greater in agonist-induced platelet aggregation with α = 0.05 and a within-subject SD of 5.5%.

### Statistical analysis

Differences from baseline in study outcomes were analyzed using a linear mixed model for a 2-period crossover study with 2, 3, or 4 repeated measures posttreatment. The model included volunteers as random factors and treatment, time and sequence as fixed effects, and was adjusted for sex (male/female), age (30–42 y/43–55 y), and BMI (20–24.9 and 25–30). Incremental AUC (iAUC) was calculated using the trapezoid rule, and a linear mixed model was conducted to compare treatment effects, with a volunteer as a random effect, treatment and sequence as fixed effects, and adjustments for sex, age, and BMI. Model assumptions were assessed through diagnostic plots of residuals. The strengths of the correlations between polyphenol metabolites and biological characteristics were assessed by Pearson’s correlation coefficient or Spearman’s correlation coefficient where appropriate. Significantly associated variables were entered into a multivariate regression model, and all variables with *P* values <0.05 were subsequently incorporated into a stepwise multivariate regression model, in which parameters of F ≤ 0.05 were entered, and F ≥ 0.10 were removed to identify independent predictors of selected CVD risk markers. Results are expressed as means ± SEMs. Given that there were 2 primary outcomes (FMD and platelet aggregation), a Bonferroni correction was applied such that a *P* value of <0.025 was considered significant. The study included 8 secondary outcomes and analysis of multiple plasma and urinary metabolites, which were exploratory rather than confirmatory data and were therefore unadjusted. Statistical analyses were performed using SPSS 28 (IBM Corporation).

## Results

**FIGURE 2:**
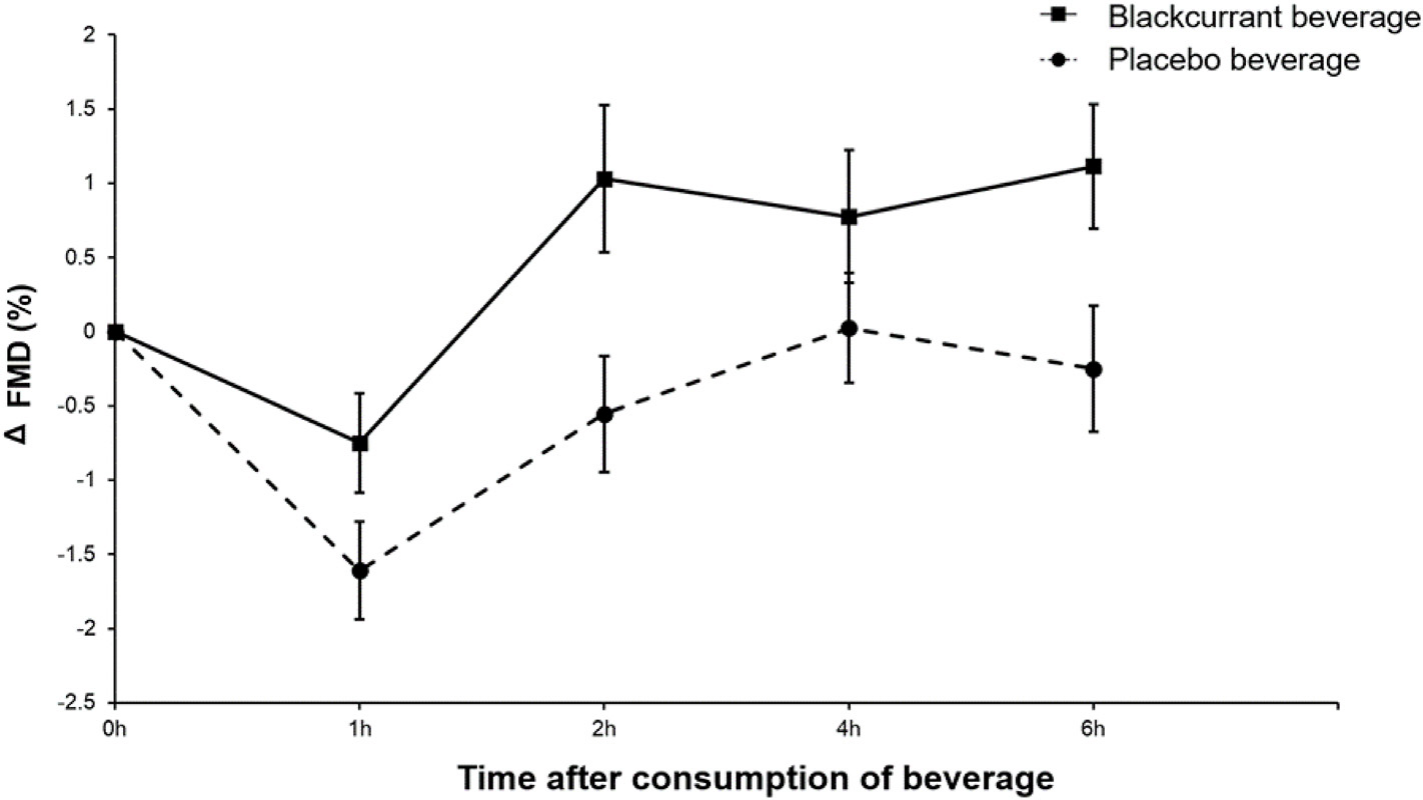
Changes from baseline in FMD after consumption of the blackcurrant beverage or the placebo together with a high-fat breakfast. Data are mean ± SEM (*n* = 22). Volunteers consumed the test beverage together with a high-fat, low-flavonoid breakfast (52.3 g fat), followed by a medium-fat lunch (30 g fat) at 3 h after the first consumption. Differences from baseline in FMD values were analyzed using a linear mixed model with treatment (2 treatments: blackcurrant compared with placebo beverage) and time (4 levels) as factors and adjusting for sex, age, and BMI. The main effects for treatment and time were significant (both *P* < 0.000001), but there was no significant treatment × time interaction (*P* = 0.425). BMI, body mass index; FMD, flow-mediated dilation; SEM, standard error of the mean.

**FIGURE 3:**
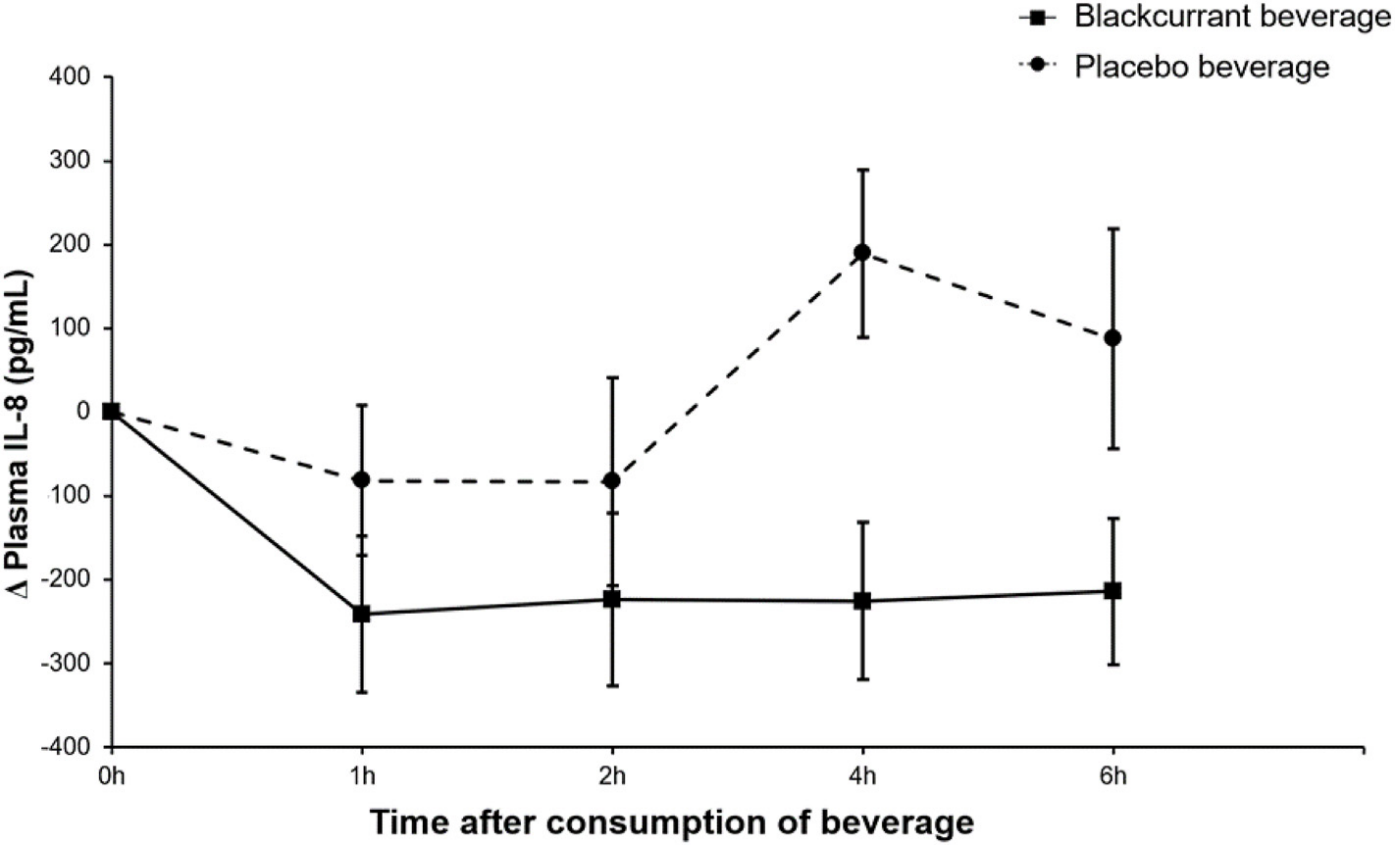
Changes from baseline in IL-8 concentrations after consumption of the blackcurrant beverage or placebo together with a high-fat breakfast. Data are mean ± SEM (*n* = 23). Volunteers consumed the test beverage together with a high-fat, low-flavonoid breakfast (52.3 g fat), followed by a medium-fat lunch (30 g fat) at 3 h after the first consumption. Differences from baseline in IL-8 concentrations were analyzed using a linear mixed model with treatment (2 treatments: blackcurrant compared with placebo beverage) and time (4 levels) as factors, adjusting for sex, age, and BMI. The treatment effect was significant (*P* < 0.001 unadjusted), but there was no significant effect of time and no treatment *×* time interaction (*P* = 0.26 and *P* = 0.33, respectively, unadjusted). BMI, body mass index; IL-8, interleukin-8; SEM, standard error of the mean.

Twenty-three subjects completed both study visits (Figure 1). There were 3 adverse events, 2 after consumption of the blackcurrant beverage and 1 after consumption of the placebo beverage (vomiting posttreatment during the study day and in 1 case, this was coupled with an uncomfortable cannula), and these subjects were withdrawn from the study. No serious adverse events were reported. Baseline characteristics of subjects who completed the study are presented in Table 1. Volunteers were healthy, normal- or overweight, and without additional CVD risk factors. There were no significant differences in baseline measures of FMD (Figure 2), platelet aggregation, SBP, DBP, DVP-SI, DVP-RI, PDEVs, or EDEVs between placebo and intervention days (Table 3), but baseline IL-8 concentration was significantly higher on days where the blackcurrant beverage was consumed (paired t-test, 2230 pg/mL compared with 1913 pg/mL; *P* < 0.01 unadjusted; Figure 3). Baseline concentrations of polyphenols and metabolites were very low, and there were no significant differences, except for baseline urinary delphinidin-3-rutinoside concentration, which was significantly higher on days when the blackcurrant beverage was consumed (paired t-test, 0.0004 nmol compared with 0.0001 nmol; *P* < 0.05 unadjusted).

**TABLE 3:** Mean subject baseline and change from baseline for vascular function and inflammatory parameters.

	Baseline	Change from baseline				Treatment effect	Treatment *×* time interaction
		1 h	2 h	4 h	6 h	*P* value	
FMD (mm)							
Placebo	7.31 ± 0.51	–1.61 ± 0.00	–0.82 ± 0.02	0.02 ± –0.01	–0.25 ± 0.08	*P* < 0.000001	0.425
Blackcurrant	7.20 ± 0.51	–0.75 ± 0.02	0.69 ± 0.12	0.77 ± 0.10	1.11 ± 0.22		
SBP (mm Hg)							
Placebo	113.1 ± 1.9	0.1 ± 0.9	–0.1 ± 1.0	3.1 ± 1.2	1.5 ± 1.0	0.015	0.806
Blackcurrant	113.7 ± 2.3	–1.0 ± 1.0	–0.8 ± 1.3	0.9 ± 1.2	0.2 ± 1.3		
DBP (mm Hg)							
Placebo	65.2 ± 1.6	–3.8 ± 0.6	–2.9 ± 0.6	–3.3 ± 0.8	–2.7 ± 0.7	0.054	0.997
Blackcurrant	65.5 ± 1.6	–4.5 ± 0.7	–3.7 ± 0.6	–4.0 ± 0.8	–3.6 ± 0.9		
DVP-SI							
Placebo	5.9 ± 0.7	/	0.3 ± 0.2	0.2 ± 0.1	0.5 ± 0.1	0.314	0.951
Blackcurrant	5.9 ± 0.6	/	0.4 ± 0.2	0.4 ± 0.2	0.7 ± 0.3		
DVP-RI							
Placebo	65.2 ± 1.2	/	–5.5 ± 2.2	–10.0 ± 2.4	–6.8 ± 2.5	0.158	0.907
Blackcurrant	65.8 ± 1.1	/	–6.6 ± 2.0	–12.2 ± 2.4	–9.7 ± 2.5		
PDEVs (per *μ*L PPP)							
Placebo	1501 ± 103.9	/	–171.1 ± 77.1	–139.8 ± 123.8	/	0.905	0.635
Blackcurrant	1482 ± 102.2	/	–118.5 ± 63.5	–171.9 ± 83.9	/		
EDEVs (per *μ*L PPP)							
Placebo	4536 ± 238.1	/	–713.4 ± 214.5	–789.4 ± 202.0	/	0.222	0.981
Blackcurrant	4457 ± 217.6	/	–497.8 ± 182.4	–582.0 ± 178.9	/		

Data are means ± SEMs (*n* = 23). Differences from baseline in study outcomes were analyzed using a linear mixed model for a 2-period crossover study. The model included volunteers as random factors and treatment, time and sequence as fixed effects, and was adjusted for sex (male/female), age (30–42 y/43–55 y), and BMI (20–24.9 and 25–30).

Abbreviations: BMI, body mass index; DBP, diastolic blood pressure; DVP, digital volume pulse; EDEV, endothelium-derived extracellular vesicle; FMD, flow-mediated dilatation; PDEV, platelet-derived extracellular vesicle; PPP, platelet-poor plasma; RI, reflection index; SBP, systolic blood pressure; SEM, standard error of the mean; SI, stiffness index.

### FMD

Baseline brachial artery diameter did not significantly change at the initiation of occlusion. The intervention demonstrated significant main effects of both treatment and time on change from baseline for FMD, but no time ∗ treatment interaction, indicating that the change in FMD followed a similar pattern in both groups (linear mixed model: *P* < 0.000001; Figure 2 and Table 3). Notably, the iAUC was significantly greater for the blackcurrant group than the placebo (Table 4), suggesting that the blackcurrant beverage led to a cumulative improvement in FMD over the 6 h postprandial period.

**TABLE 4:** Vascular function, inflammatory parameters, and extracellular vesicle: difference in incremental AUC.

	△iAUC	95% CI	*P-treatment*	*P-sequence*	*P-interaction*
FMD	5.75 ± 1.32	2.99, 8.51	0.014	0.842	0.375
DVP-SI	1.4 ± 1.8	–2.3, 5.1	0.273	0.816	0.249
DVP-RI	–7.0 ± 12.9	–34.0, 19.9	0.632	0.448	0.211
SBP	–8.8 ± 5.3	–19.9, 2.3	0.165	0.083	0.271
DBP	–4.5 ± 4.3	–13.4, 4.5	0.477	0.710	0.269
ADP					
10 μM	–30.8 ± 8.7	–49.0, –12.8	<0.01	0.103	0.123
100 μM	–18.6 ± 4.1	–27.1, –10.0	<0.001	0.679	0.474
Collagen					
0.5 μg/mL	–16.4 ± 6.7	–30.6, –2.3	<0.01	0.134	0.395
1 μg/mL	–30.7 ± 9.0	–49.6, –11.9	<0.01	0.681	0.692
IL-8	–1826.8 ± 618.6	–3121.5, –532.2	0.015	0.310	0.726
PDEVs	–226.3 ± 374.6	–1010.3, 557.7	0.841	0.520	0.804
EDEVs	–52.9 ± 734.3	–1589.9, 1484.1	0.440	0.735	0.878

Data are mean differences ± SEMs (*n* = 23). Incremental AUC was calculated using the trapezoid rule, and a linear mixed model was conducted to compare treatment effects, with volunteer as a random effect, treatment and sequence as fixed effects, and adjustments for sex, age, and BMI.

### DVP

The blackcurrant beverage had no effect on the DVP-SI or the DVP-RI (Table 3), and there were no differences in iAUCs (Table 4).

### BP

The linear mixed model indicated significant main effects of both treatment and time for SBP (*P-treatment* = 0.015 and *P-time* = 0.004 unadjusted), whereas there was no significant treatment ∗ time interaction (*P* = 0.806 unadjusted; Table 3) and no significant difference in iAUCs (Table 4). Regarding DBP, there was a trend for a main effect of treatment (*P-treatment* = 0.054 unadjusted; Table 3), but the iAUCs were not significantly different (Table 4), there was no significant effect of time (*P* = 0.29 unadjusted) and no treatment ∗ time interaction (*P* = 0.997 unadjusted).

### Agonist-induced platelet aggregation

There was a significant inhibitory effect of the blackcurrant beverage on platelet aggregation stimulated by both ADP (10 μM and 100 μM) and 1 μg/mL collagen (*P-treatment* = 0.00003, 0.001, and 0.0005 unadjusted, respectively, Table 5) and a trend in response to 0.5 μg/mL collagen concentration (*P-treatment* = 0.069 unadjusted, Table 5). There was no significant treatment ∗ time interaction for either agonist at either concentration, but there were significant differences in iAUCs for ADP at both 10 μM and 100 μM and for collagen at both 0.5 μg/mL and 1 μg/mL (TABLE 4, TABLE 5), suggesting a cumulative reduction in platelet aggregation over the course of the postprandial period, particularly when ADP was used as an agonist.

**TABLE 5:** Mean subject baseline and change from baseline for ex vivo ADP- and collagen-induced platelet aggregation.

	Baseline		Change from baseline				Treatment effect	Treatment *×* time interaction
			Placebo		Blackcurrant		*P* value	
	Placebo	Blackcurrant	2 h	4 h	2 h	4 h		
ADP (%)								
10 μM	27.5 ± 5.3	35.6 ± 6.0	1.6 ± 2.0	–3.4 ± 1.6	–10.0 ± 2.8	–11.0 ± 3.7	0.00003	0.458
100 μM	74.1 ± 1.8	76.7 ± 1.1	2.0 ± 1.4	–2.5 ± 2.0	–6.3 ± 2.3	–5.6 ± 1.4	0.001	0.151
Collagen (%)								
0.5 *μ*g/mL	44.6 ± 5.6	35.3 ± 5.9	–0.1 ± 2.3	–5.1 ± 3.2	–6.9 ± 2.0	–9.1 ± 4.2	0.069	0.633
1 *μ*g/mL	72.5 ± 2.5	74.7 ± 1.7	0.4 ± 2.2	–2.1 ± 1.9	–11.0 ± 3.2	9.9 ± 3.4	0.0005	0.511

Data are means ± SEMs (*n* = 21). Platelet aggregation was assessed after 5 min exposure to an agonist. The extent of platelet aggregation was not different at baseline for either agonist at either concentration. Differences from baseline in study outcomes were analyzed using a linear mixed model for a 2-period crossover study. The model included volunteers as random factors and treatment, time and sequence as fixed effects, and was adjusted for sex (male/female), age (30–42 y/43–55 y), and BMI (20–24.9 and 25–30). iAUCs for ADP were significantly lower in the blackcurrant group (101.4 ± 51.1 compared with 114.6 ± 50.6 at 10 *μ*M and 288.4 ± 17.0 compared with 298.0 ± 14.9 at 100 *μ*M; *P* = 0.001 and 0.004 respectively, unadjusted). iAUCs for collagen were also significantly lower in the blackcurrant group (118.5 ± 55.2 compared with 173.3 ± 52.6 at 0.5 *μ*g/mL and 267.1 ± 33.7 compared with 288.6 ± 26.8 at 1 *μ*g/mL; *P* = 0.04 and 0.002 respectively, unadjusted).

Abbreviations: ADP, adenosine diphosphate; BMI, body mass index, CI, confidence interval; DBP, diastolic blood pressure; DVP, digital volume pulse; EDEV, endothelium-derived extracellular vesicle; FMD, flow-mediated dilatation; iAUC, incremental area under the curve; IL-8, interleukin-8; PDEV, platelet-derived extracellular vesicle; RI, reflection index; SBP, systolic blood pressure; SEM, standard error of the mean; SI, stiffness index.

### IL-8

The change from baseline in IL-8 concentration was significantly different over the course of the study day after consumption of the blackcurrant beverage compared with the placebo, with the postprandial increase in IL-8 being completely absent in the blackcurrant group (*P-treatment* = 0.0012 unadjusted, Figure 3). This was also evident as a significantly altered iAUC (Table 4).

### EVs

The blackcurrant beverage had no effect on the numbers of EDEVs or PDEVs (Table 3) or on iAUCs (Table 4).

### Parent anthocyanins, anthocyanin conjugates, and phenolic metabolites changes in plasma and urine

**FIGURE 4:**
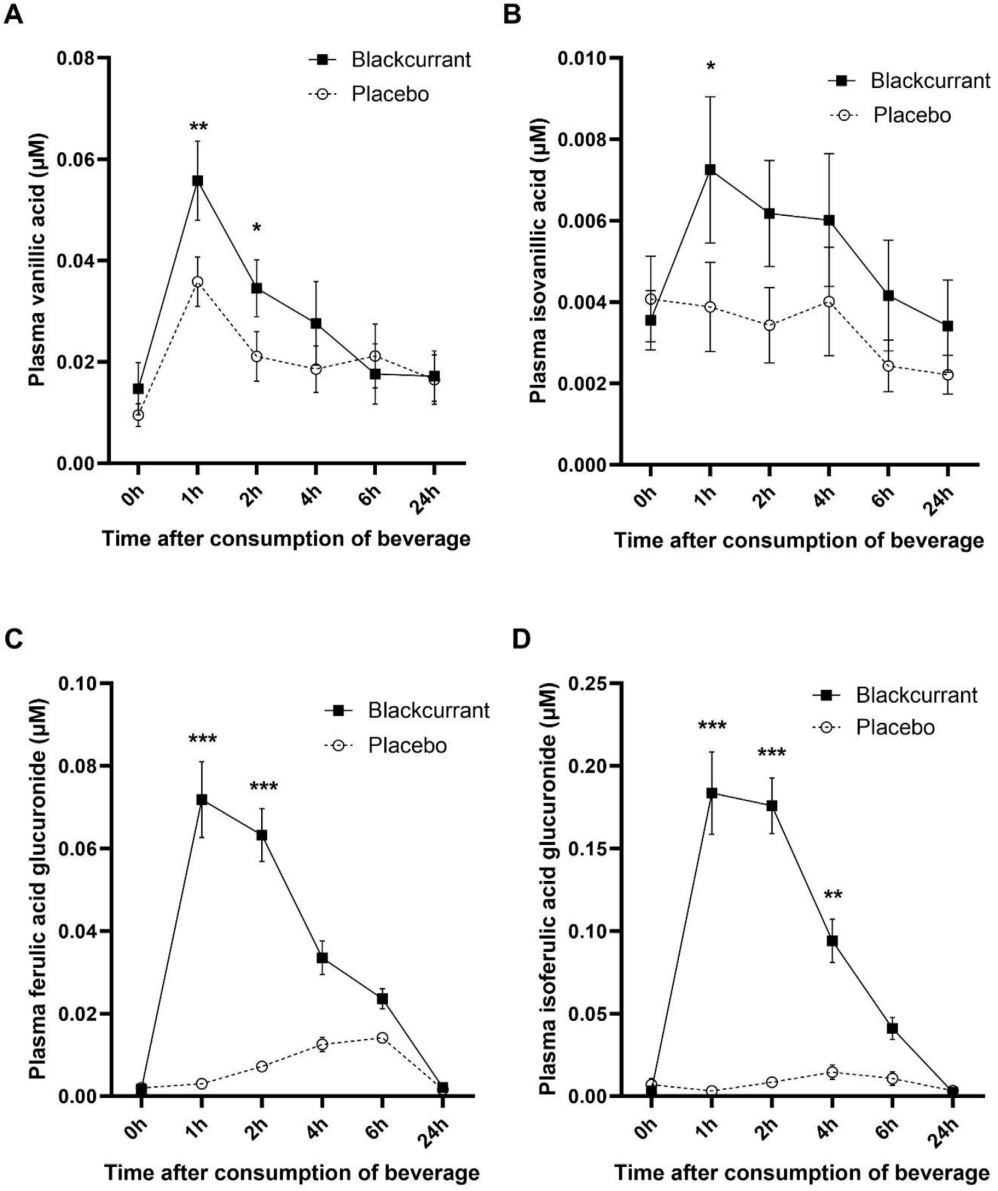
Changes in plasma concentrations of anthocyanin conjugates and phenolic metabolites after consumption of the blackcurrant beverage or placebo together with a high-fat breakfast. Data are mean ± SEM (*n* = 21). Volunteers consumed the test beverage together with a high-fat, low-flavonoid breakfast (52.3 g fat), followed by a medium-fat lunch (30 g fat) at 3 h after the first consumption. Data were analyzed by using a linear mixed model with treatment (2 treatments: blackcurrant compared with placebo beverage) and time (4 levels) as factors, adjusting for sex, age, and BMI, and post hoc analyses were conducted by using a Bonferroni multiple-comparisons test. Plasma concentrations of (A) vanillic acid at 1 h and 2 h and (B) isovanillic acid at 1 h after the consumption of the blackcurrant drink were significantly increased compared with the placebo. Plasma concentrations of (C) ferulic acid glucuronide at 1 h and 2 h and (D) isoferulic acid glucuronide at 1 h, 2 h, and 4 h after the consumption of the blackcurrant drink were significantly elevated compared with the placebo. (E) Plasma concentrations of cyanidin glucuronide were significantly increased at 1 h and 2 h postconsumption of the blackcurrant drink compared with the placebo. ∗, ∗∗, ∗∗∗ Significantly different from the placebo drink at the specified time point: ∗*P* < 0.05, ∗∗*P* < 0.01, ∗∗∗*P* < 0.001. BMI, body mass index; SEM, standard error of the mean.

Following analysis by ultra-performance liquid chromatography-MS/MS, 15 compounds in plasma and 30 compounds in urine were identified as parent anthocyanins, anthocyanin conjugates, and phenolic metabolites derived from the blackcurrant beverage. Total phenolics in plasma peaked at 2–4 h post consumption and amounted to 3.1 ± 0.14 μM total phenolics over the 24 h period. Plasma concentrations of vanillic acid and ferulic acid glucuronide were significantly elevated at 1 h and 2 h, with a significant increase in isovanillic acid at 1 h and isoferulic acid glucuronide at 1 h, 2 h, and 4 h after consumption of the blackcurrant drink compared with the placebo (Figure 4 A–D). Plasma cyanidin glucuronide was also significantly increased at 1 h and 2 h postconsumption (Figure 4E). There was no significant increase in plasma concentrations of any other anthocyanin conjugates or phenolic metabolites, and parent anthocyanins were not detectable in the pre or postintake plasma (data not shown).

Excretion of urinary total phenolic metabolites increased between 0 h and 1 h following consumption of the blackcurrant beverage and amounted to 56 ± 18.1 μmol total phenolics over the 24 h period. Urinary excretion of ferulic acid, ferulic acid sulfate, and ferulic acid glucuronide with their isomers, delphinidin-3-glucoside, and phloroglucinaldehyde, was significantly increased between 0 h and 1 h; all except isoferulic acid and ferulic acid glucuronide with its isomer remained significantly increased at 1–2 h. Isoferulic acid, ferulic acid glucuronide, delphinidin-3-glucoside, delphinidin-3-rutinoside, and phloroglucinaldehyde were significantly increased 2–4 h after consumption of the blackcurrant drink compared with the placebo (Supplemental Figure 1). Over the whole 24 h period following consumption of the blackcurrant beverage, there was significantly greater urinary excretion of ferulic acid sulfate, ferulic acid glucuronide and its isomer, phloroglucinaldehyde, cyanidin-3-glucoside, delphinidin-3-glucoside and delphinidin-3-rutinoside than the placebo (Supplemental Figure 2A). There was no significant increase in any other parent anthocyanins, anthocyanin conjugates, or phenolic metabolites, including hippuric acid, 4-hydroxybenzaldehyde, or 3-hydroxybenzoic acid (Supplemental Figure 2B) over the whole 24 h period following consumption of the blackcurrant beverage.

### Plasma phenolic metabolites and anthocyanin conjugates are associated with FMD, BP, inhibition of platelet aggregation, and IL-8

**FIGURE 5:**
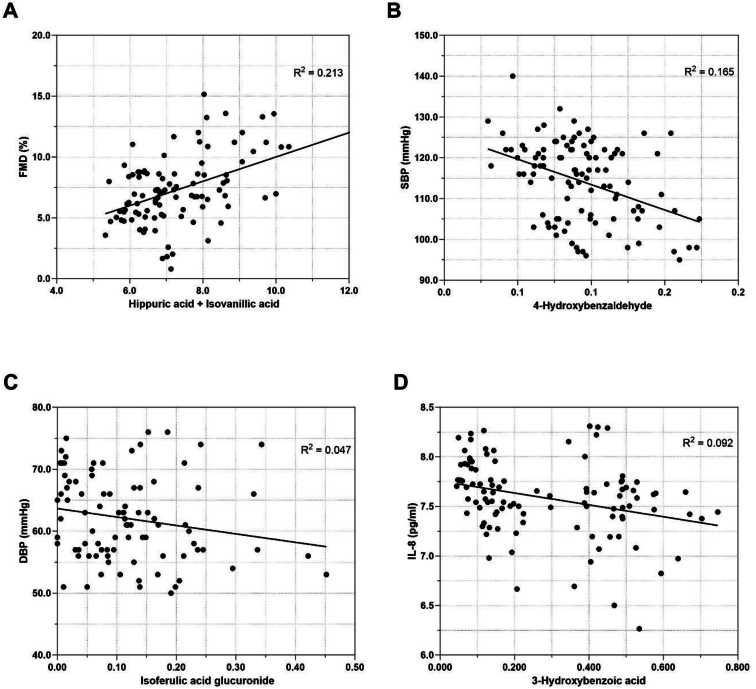
Associations between plasma phenolics metabolites with vascular function and inflammatory parameters after the consumption of the blackcurrant beverage together with a high-fat breakfast. Data are analyzed by using a stepwise multivariate regression model to identify independent predictors of selected CVD risk markers. (A) Plasma isovanillic acid and hippuric acid concentrations independently explained 21.3% of the variance in FMD; (B) 4-hydroxybenzaldehyde concentration explained 16.5% of the variance for SBP; (C) isoferulic acid glucuronide concentration explained 4.7% of the variance for DBP; (D) 3-hydroxybenzoic acid concentration explained 9.2% of the variance for IL-8. CVD, cardiovascular disease; DBP, diastolic blood pressure; FMD, flow-mediated dilation; IL-8, interleukin-8; SBP, systolic blood pressure.

The plasma metabolites most significantly associated with FMD, one of the primary outcomes of the trial, were hippuric acid (r = 0.410, *P* < 0.001) and isovanillic acid (r = 0.354, *P* < 0.001), both emerging as strong independent predictors of FMD and explaining 21.3% of the variance (Figure 5A; Table 6). Baseline concentrations of hippuric acid and isovanillic acid were also significantly correlated with baseline FMD (r = 0.319, *P* = 0.045; r = 0.452, *P* = 0.003, respectively), with isovanillic acid predicting 27.6% of the variance for baseline FMD. However, although there was a significant increase in the plasma concentration of isovanillic acid following consumption of the blackcurrant beverage (Figure 4B), there was no significant increase in hippuric acid, nor was there an increase in urinary concentration, although it was the most abundant plasma metabolite in both plasma and urine (Supplemental Figures 2B and 3A and B). Plasma concentrations of hydroxybenzaldehyde, hydroxybenzoic, vanillic, ferulic acid, and cyanidin glucuronide were negatively associated with SBP and/or DBP (Table 6). Stepwise regression analysis suggested that concentrations of 4-hydroxybenzaldehyde explained 16.5% of the variance for SBP (Figures 5B), and isoferulic acid glucuronide explained 4.7% of the variance for DBP (Figures 5C). The latter corresponded with a significant increase in the plasma concentration of isoferulic acid glucuronide after consumption of the blackcurrant beverage (Figure 4D), but there was no such increase in the plasma concentration or urinary excretion of 4-hydroxybenzaldehyde (Supplemental Figures 2B and 3C and D). Higher plasma concentration of isovanillic acid was significantly associated with the inhibition of platelet aggregation, but this was limited to samples stimulated by 10 μM ADP (r = –0.302, *P* = 0.022). Plasma concentrations of the hydroxybenzoic acid family were associated with plasma concentrations of IL-8 (Table 6), with 3-hydroxybenzoic acid explaining 9.2% of the variance (Figure 5D), but there was no detectable increase in this metabolite, either in the plasma or in the urine, after consumption of the blackcurrant beverage compared with the placebo (Supplemental Figures 2B and 3E and F).

**TABLE 6:** Associations between plasma anthocyanin conjugates and phenolics metabolites with vascular function, platelet function, and inflammatory parameters after consumption of the blackcurrant beverage.

		FMD	SBP (mm Hg)	DBP (mm Hg)	ADP (10 *μ*M)	ADP (100 *μ*M)	Collagen (1 μg/mL)	IL-8
Phenolics metabolites								
4-hydroxybenzaldehyde	*r*	.171	–.321∗∗	–.173	–.034	–.003	–.001	–.096
	*p*	.090	<.001	.077	.799	.981	.995	.330
Hippuric acid	*r*	.410∗∗	–.174	–.073	–.134	.045	.094	–.014
	*p*	<.001	.076	.462	.321	.739	.487	.885
2-hydroxybenzoic acid	*r*	.126	–.170	–.041	–.064	–.153	–.020	–.301∗∗
	*p*	.216	.082	.676	.637	.255	.883	.002
3-hydroxybenzoic acid	*r*	.042	–.239∗	–.135	–.222	–.071	–.078	–.382∗∗
	*p*	.684	.015	.173	.100	.605	.568	<.001
4-hydroxybenzoic acid	*r*	.067	–.219∗	–.123	–.167	–.066	–.062	–.367∗∗
	*p*	.510	.025	.213	.219	.631	.649	<.001
3,5-dihydroxybenzoic acid	*r*	.088	–.165	–.215∗	–.033	–.008	.092	–.189
	*p*	.389	.092	.028	.806	.953	.496	.053
Vanillic acid	*r*	.051	–.124	–.223∗	–.007	–.008	–.159	–.093
	*p*	.613	.207	.022	.960	.951	.238	.344
Isovanillic acid	*r*	.354∗∗	.132	.000	–.302∗	–.052	–.165	–.010
	*p*	<.001	.179	.998	.022	.701	.219	.919
Ferulic acid glucuronide	*r*	–.104	–.165	–.220∗	–.089	–.239	–.127	–.100
	*p*	.307	.092	.024	.511	.074	.346	.311
Isoferulic acid glucuronide	*r*	–.056	–.104	–.217∗	–.095	–.195	–.064	–.117
	*p*	.581	.290	.026	.480	.146	.637	.233
Anthocyanin conjugates								
Cyanidin aglycone	*r*	.099	–.018	–.124	–.138	–.078	–.146	.032
	*p*	.331	.857	.207	.307	.567	.277	.744
Cyanidin glucuronide	*r*	–.120	–.186	–.239∗	–.046	–.185	–.135	–.110
	*p*	.239	.057	.014	.733	.168	.318	.263
Cyanidin methyl glucuronide	*r*	–.013	.011	–.012	–.096	.027	–.037	.162
	*p*	.897	.910	.907	.476	.843	.785	.098

Pearson’s correlation coefficient or Spearman’s correlation coefficient was conducted to examine the associations between plasma polyphenol metabolites and trial outcomes. ∗. Correlation is significant at the 0.05 level (2-tailed). ∗∗. Correlation is significant at the 0.01 level (2-tailed).

Abbreviations: ADP, adenosine diphosphate; DBP, diastolic blood pressure; FMD, flow-mediated dilation; IL-8, interleukin-8; SBP, systolic blood pressure.

## Discussion

This RCT demonstrated a significant cumulative improvement in the primary outcome, vascular function, following consumption of an anthocyanin-rich blackcurrant beverage compared with a matched placebo in conjunction with a postprandial challenge provided by a high-fat meal. There was a trend for an inhibitory effect of the blackcurrant beverage on agonist-induced platelet aggregation and significant effects on the secondary outcomes, SBP and IL-8, although these were exploratory and not adjusted for multiple testing. Plasma analysis demonstrated that specific anthocyanin metabolites, rather than the parent anthocyanins, were likely to be the bioactive components, with plasma concentrations of hippuric acid and isovanillic acid notably being strong independent predictors of FMD and 4-hydroxybenzaldehyde and isoferulic acid glucuronide being predictors of SBP and DBP.

The novel observation of a beneficial cumulative effect of blackcurrant-derived anthocyanins compared with a matched placebo on FMD during the 6 h postprandial period following a high-fat meal in the current study is in line with reports demonstrating similar effects of 319–1791 mg blueberry polyphenols in healthy males [9,26], 694 mg açai polyphenols in males with overweight [27], and 320 mg purified bilberry and blackcurrant anthocyanins in adults with hypercholesterolemia [28], although there was no effect of a boysenberry beverage containing 351 mg polyphenols on FMD in a small (and almost certainly underpowered) study of 6 volunteers [29]. Given that a 1% FMD increase is associated with a 13% reduced risk of cardiovascular events [30], this could be considered clinically relevant if the acute effects were sustained long-term. Limited available data from studies using purified anthocyanins or fruit-based interventions of 2–12 wk duration overall demonstrate beneficial effects on FMD [28,29,31]. A meta-analysis on the more widely studied chocolate/cocoa flavan-3-ols indicates that improvements in FMD tend to be less pronounced after chronic intake (1.34% compared with 3.19%) [32], suggesting that the acute effects may not be sustained. However, in the current study, baseline plasma hippuric acid concentration, which is widely used as a biomarker of fruit and vegetable intake [33,34], was strongly associated with FMD, suggesting that there may be chronic effects of flavonoids on FMD. Moreover, a study combining a high-fat meal challenge with açai polyphenols reported a similar magnitude of improvement in FMD as the current study [27], and it could be argued that because the majority of the day is spent in a postprandial state and beverages are commonly consumed together with food, the study design employed in the current RCT is more physiologically relevant than those where anthocyanins are delivered in the absence of food.

Anthocyanins tend to have rapid rates of absorption, metabolism, and elimination, which is consistent with the lack of detection of parent compounds in the plasma and urinary analysis presented in this paper. In the current RCT there were significant elevations in the plasma concentrations of vanillic acid, isovanillic acid, ferulic acid glucuronide, isoferulic acid glucuronide and cyanidin glucuronide, as well as in the urinary excretion of ferulic acid, ferulic acid sulfate, ferulic acid glucuronide, phloroglucinaldehyde, cyanidin-3-glucoside, delphinidin-3-glucoside, and delphinidin-3-rutinoside following consumption of the blackcurrant beverage compared with the placebo. However, there were no significant increases in hippuric acid, 4-hydroxybenzaldehyde, and 3-hydroxybenzoic acid, either in the plasma or in the urine, after the consumption of the blackcurrant beverage. A study examining the effects of a blackcurrant drink (in the absence of a meal) containing 1029 mg anthocyanins reported an elevation of plasma vanillic acid, ferulic acid, and hippuric acid 1h after consumption [35]. Published data also suggest that plasma concentrations of vanillic acid, ferulic acid, and cyanidin glucuronide were increased following consumption of elderberry extract [36], and plasma concentrations of vanillic acid, ferulic acid, isoferulic acid, hippuric acid, and 2-hydroxybenzoic acid were elevated after consumption of blueberries [9]. The appearance of vanillic acid and ferulic acid in plasma reflects the rapid microbial and enzymatic metabolism of anthocyanins. Hydroxybenzoic acid is a key breakdown product of cyanidin and delphinidin (the major anthocyanin components of the blackcurrant beverage), and hippuric acid has been demonstrated to be a key circulating metabolite of cyanidine-3-glucoside [20]. Although vanillic and ferulic acid appears rapidly in plasma, hydroxybenzoic acid, and hippuric acid are secondary metabolites resulting from both microbial and hepatic metabolism and typically peak at 6–24 h [20]. The lack of increase in either hydroxybenzoic acid or hippuric acid may have been due to the fact that the metabolites had already peaked and returned to baseline by 24 h in the case of plasma, and the pooling of urine samples between 6 and 24 h may have rendered any changes in metabolites too small to be detectable. Significant correlations between FMD and plasma concentrations of hippuric acid and vanillic acid have been reported by 2 related studies examining the effects of blueberry drinks, albeit with lower concentrations of total anthocyanins compared with the current study (310 mg, 339 mg, and 150 mg); however, in these cases, plasma concentrations of both hippuric acid and vanillic acid were elevated after consumption of the blueberry drink compared with placebo [9,26]. The fact that isovanillic acid was strongly associated with FMD both at baseline and after consumption of the blackcurrant beverage suggests that it may have both acute and chronic effects on vascular function. In contrast, hippuric acid was only associated with FMD at baseline and may therefore exhibit longer-term effects. A chronic, rather than acute, effect would be consistent with hippuric acid being one of the final microbial metabolites of dietary polyphenol biotransformation and a biomarker of fruit and vegetable intake [33,34]. Nevertheless, it is not clear why some acute studies report elevation in plasma and urinary concentrations of hippuric acid, whereas others, including the current study, do not. Variations in the anthocyanin profile of berries may also influence the plasma biomarker profile; for example, chlorogenic acid, a precursor of hippuric acid, was identified as a candidate biomarker for blueberry but not blackcurrant consumption [37,38]. Blueberries are also notably rich in pelargonidin-3-O-glucoside, resulting in increased concentrations of hydroxybenzoic acid in plasma [39,40].

There is currently limited information regarding the effect of anthocyanins on arterial stiffness. Although it has been shown to correlate with FMD, the current RCT reports that neither DVP-SI nor DVP-RI were acutely altered by blackcurrant anthocyanins compared with the placebo. A similar lack of effect on arterial stiffness was observed following acute ingestion of 766–1791 mg blueberry polyphenols [9]. However, a cross-sectional study reported an inverse association between higher anthocyanin intake and reduced arterial stiffness [5], an 8-wk blueberry intervention reported improved arterial stiffness [41], and acute improvements in DVP have also been reported with other polyphenol classes in a few cases [42,43].

Data from this study indicate that an anthocyanin-rich blackcurrant beverage beneficially ameliorated changes in BP that occurred in response to a high-fat meal compared with the placebo, with isoferulic acid glucuronide and 4-hydroxybenzaldehyde being independent predictors, although only the former was significantly elevated in the plasma, with no significant change in either plasma concentration or urinary excretions of 4-hydroxybenzaldehyde after consumption of the blackcurrant beverage compared with the placebo. Several previous RCTs report no acute effects following the consumption of blueberry beverages [9,26], a blueberry bun [26], or an açai smoothie [27]. These studies tested lower amounts of anthocyanins (ranging from 196–724 mg), suggesting that high doses might be required to modulate BP. However, if the active compounds are isoferulic acid glucuronide and 4-hydroxybenzaldehyde, the flavonoid profile of the fruit and the metabolism of the anthocyanins may be key to biological activity. Although the current study is suggestive of effects on BP, they represent exploratory results for a secondary outcome unadjusted for multiple comparisons and are therefore associated with an inflated risk of type 1 errors. Nevertheless, the identification of potential bioactive components provides a basis for future investigation of pure compounds or specifically enriched products and validation of the data.

To our knowledge, this is the first RCT investigating the acute effect of an anthocyanin-rich intervention on platelet function. Consumption of the blackcurrant beverage significantly inhibited ex vivo ADP- and collagen-induced platelet aggregation compared with the matched placebo. Only 2 previous studies examined the acute antiplatelet action of anthocyanin-rich products, but both focused on acute-on-chronic effects. In a small, uncontrolled study with 10 volunteers, consumption of purple grape juice containing 1017 mg total polyphenols significantly inhibited collagen-induced platelet aggregation, but there were no effects on ADP- or thrombin-induced platelet aggregation, indicating that only the collagen-induced signaling pathway was modulated [44]. In an RCT with 35 healthy males, a smaller dose of 400 mg total polyphenols, delivered in the form of a wine extract capsule, had no significant effect on ADP- or collagen-induced platelet aggregation at 1.75 h posttreatment, but there was a trend for inhibited platelet aggregation after consumption of lunch and another wine extract capsule [45]. However, identification of the bioactive compounds was not possible; similarly, in the current study, there was only a minor negative association between platelet aggregation and plasma isovanillic acid concentration. Chronic administration of anthocyanin-rich products has also been shown to improve platelet function in some [[46], [47], [48]] but not all studies [[49], [50], [51]]. Direct comparisons between studies are virtually impossible as a number of different methods are used for platelet function assessment, and important details regarding anthocyanin dose and/or composition are often missing. The present study used the gold standard technique, ex vivo platelet aggregation in PRP, and this method has been shown to be associated with coronary artery disease mortality [11] and previous myocardial infarction [52].

The effects of the blackcurrant beverage compared with the matched placebo on plasma concentration of IL-8 are suggestive of its potential to reduce inflammation and neutrophil recruitment and improve endothelial and vascular repair. However, there is limited published information relating to the effects of anthocyanins on the plasma concentration of IL-8. There was no effect of 375 g blueberry 1 h prior to 2.5 h of treadmill running on plasma IL-8 or of 250 g/d of whole blueberries for 6 wk [53] or of freeze-dried strawberry powder for 3 wk [54]. On the contrary, consumption of 60 g/d freeze-dried black raspberry powder (1669 mg total anthocyanins) by patients with colorectal cancer for 3 wk [55] or Medox capsules containing 300 mg total anthocyanins isolated from bilberries by healthy subjects [18] decreased plasma IL-8 concentration. It is notable that IL-8 concentrations are dependent on the menstrual cycle, and although potential cycle-induced effects were controlled for in the female subjects in the current study, it was not possible to start them all at the same phase in their cycle.

It has previously been demonstrated that improvements in FMD in patients with coronary artery disease after 4 wk of consumption of a cocoa drink were paralleled by a decrease in the number of circulating EDEVs [16]. However, the current study demonstrated no significant effect of the blackcurrant beverage compared with the matched placebo on the number of circulating EVs in healthy subjects. The potential importance of health status was suggested in another chronic cocoa study, where consumption of a daily cocoa bar reduced circulating numbers of EDEVs in volunteers with overweight or obesity, who had initial higher values, but not in volunteers with normal weight [17]. To our knowledge, the present RCT is the first study to investigate the acute postprandial effect of an anthocyanin-rich intervention on circulating EDEVs and PDEVs, and thus, comparable data are lacking.

The strengths of this study include the fact that it has a suitably powered crossover design with a blackcurrant drink containing amounts of anthocyanins that are achievable through the diet (711 mg anthocyanins, corresponding to 120 g fresh blackcurrants) and a macronutrient- and micronutrient-matched control drink. Moreover, the design includes the provision of sequential meals, which replicate a real-life scenario. Results for secondary outcomes, though suggestive, are unadjusted for multiple comparisons, carry a risk of inflated type 1 errors, and should therefore be interpreted as exploratory. This paper reports, in detail, changes in the plasma and urinary concentrations of anthocyanins and phenolic metabolites after consumption of both the blackcurrant and placebo beverage and evaluates likely bioactive components associated with the improvements in vascular, platelet, and inflammatory functions. Although causal relationships cannot be established, the data provide clear directions for further investigation and a significant advance on previous knowledge. A potential limitation is that this was an acute study, and the effects of longer-term consumption and mechanisms of action are not yet clear.

In conclusion, the present study demonstrates, to our knowledge, for the first time, that an anthocyanin-rich blackcurrant beverage can beneficially ameliorate changes in vascular function and markers of CVD risk in response to a high-fat meal compared with a matched placebo. Improvements were observed in FMD, platelet function, IL-8 concentrations, and some aspects of SBP, with plasma concentrations of hippuric acid and isovanillic acid predicting FMD and 4-hydroxybenzaldehyde and isoferulic acid glucuronide predicting SBP and DBP. The data suggest that anthocyanins can attenuate the deleterious effects of a dietary fat challenge and could represent an effective mitigating factor for CVD prevention.

## Author contributions

The authors’ responsibilities were as follows – AMA, JPES, PY: designed the study; AMA, RZ, KA, DK, ISI: conducted the human study; GS, GC: performed the polyphenol and metabolite analysis of plasma and urine samples; TB-M: developed the methodology for the extracellular vesicles measurements; HA: performed the polyphenol analysis of the study beverages under supervision of PAK; AMA, RZ, GS: analyzed data and performed statistical analyses; AMA, RZ, GS, PY: wrote the manuscript; PY: had primary responsibility for final content; and all authors: read and approved the final manuscript.

## Data availability

Data described in the manuscript will be made publicly and freely available without restriction from the University of Reading Research Data Archive at https://doi.org/10.17864/1947.001321.

## Funding

Supported by the Biotechnology and Biological Sciences Research Council (BBSRC) through a BBSRC CASE studentship with GlaxoSmithKline as an industrial sponsor and by Institute Strategic Programme Grants "Food Innovation and Health" (BB/R012512/1) and its constituent project BBS/E/F/000PR10343 and "Food Microbiome and Health" (BB/X011054/1) and its constituent project BBS/E/QU/230001D. HA was funded by the Newton-Mosharafa Scholarship Fund from the Egyptian Ministry of Higher Education (Cultural Affairs and Mission Sector), the British Council, and the British Embassy in Egypt.

## Conflict of interest

PY reports financial support was provided by Biotechnology and Biological Sciences.

Research Council and GlaxoSmithKline. PK reports financial support was provided by Biotechnology and Biological Sciences Research Council. GS reports employment relationships with both GlaxoSmithKline Consumer Health and Haleon plc that includes employment. HA reports financial support was provided by Egyptian Ministry of Higher Education and British Council. All other authors report no conflicts of interest.
