# Supplementary material for: Acute Effects of an Anthocyanin-Rich Blackcurrant Beverage on Markers of Cardiovascular Disease Risk in Healthy Adults: A Randomized, Double-Blind, Placebo-Controlled, Crossover Trial
Source: J Nutr. 2025 May 23;155(7):2275–89. doi: 10.1016/j.tjnut.2025.05.017 (PMC12308090; doi:10.1016/j.tjnut.2025.05.017)
Supplement: Multimedia component 1 [file mmc1.docx]

**Acute effects of an anthocyanin-rich blackcurrant beverage on markers of cardiovascular disease risk in healthy adults: a randomized, double-blind, placebo-controlled, crossover trial**

Amini A.M. *et al.*

**Online Supplementary Material**

# **Supplementary Methods**

### **Quantifications of polyphenols in the test beverage**

*Quantification of anthocyanins by HPLC/MS*

100 mg of the extract powder was extracted by dissolving in 50 ml acidified aqueous methanol (water: methanol: formic acid 70: 28: 2) and then centrifuged at 13000 rpm for 5 min. 20µl of supernatant was injected onto an HPLC/MS system in which chromatographic separation was acheived using a reverse phase Kinetex XB-C18 HPLC column (100× 4.6 mm; particle size 2.6 μm) at 40°C using 5% aqueous formic acid (eluent A) and 5% formic acid in acetonitrile (eluent B). A wide range of UV-Vis wavelengths were used for monitoring the eluents and the anthocyanins detection was achieved at 520 nm. The eluent was then introduced into a single quadrupole mass spectrometer in positive ion polarization mode. The identification and peak assignment of anthocyanins in the extract were based on a comparison of their retention times and mass spectral data with three reference standards (cyanidin-3-O-glucoside, delphinidin-3-O-glucoside and delphinidin-3-O-rutinoside) and blackcurrant published data [1], and the quantification was performed using external standards (cyanidin-3-O-glucoside, delphinidin-3-O-glucoside and delphinidin-3-O-rutinoside). To establish the standard curve, a range of working concentrations (0 to 150 µg/ml) were freshly prepared by diluting with acidified methanolic aqueous solution and dimethyl sulfoxide (DMSO) was added to get them all contain the same quantity of DMSO. Three replicates from the powder were extracted and analyzed.

*Quantification of procyanidins by HPLC/MS*

50 mg of the extract powder was extracted by dissolving in 1 ml 70% methanol (60°C), heated at 60°C for 30 min, and then centrifuged for 5 min at 17000g. 2µl of extract was injected onto an HPLC system connected to a fluorescence detector in which the chromatographic separation was conducted using a Phenomenex Luna Hilic HPLC column (150 x 2.0 mm; particle size 3 μm) at 35°C using 2% acetic acid in acetonitrile (eluent A) and 2% acetic acid, 95% methanol and 3% water as eluent B. Fluorescence detection which can specifically detect flavanols and polymeric flavanols (procyanidins) was used to monitor the elution of compounds. The identification and peak assignment of procyanidins in the samples were based on a comparison of their retention times with standard epicatechin and the retention times of a well characterised mixture of apple procyanidins [2-3]. The quantification was performed an external standard curve with epicatechin as the reference standard. Due to a lack of authentic reference standards of all individual polymeric flavanols, the procyanidins were measured relative to an epicatechin standard and calculated using relative response factor that are obtained from apple procyanidins standards [2]. Because the signals were very small for most of the putative proanthocyanidins, a number of peaks for each degree of polymerization were grouped together using the range of retention times that were observed for the isolated apple procyanidin fractions [2]. The total peak area then was used to quantify the concentration relative to epicatechin using the relative response factor that corresponded with the equivalent apple procyanidin. To establish the standard curve, a range of working concentrations of epicatechin (0 to 50 µg/ml) were freshly prepared by diluting with 70% methanol and DMSO was added to get them all contain the same quantity of DMSO.

### **Quantifications of anthocyanin and phenolic metabolites in plasma and urine**

Identification and quantification of anthocyanin and phenolic metabolites was conducted by UPLC-MS/MS following solid phase extraction.

*Materials*

Cyanidin-3-glucoside, cyanidin-3-rutinoside, delphinidin-3-glucoside, delphinidin-3-rutinoside were obtained from Extrasynthese; vanillic acid, isovanillic acid, syringic acid, 4-hydroxybenzaldehyde, hippuric acid, 2-hydroxybenzoic acid, 3-hydroxybenzoic acid, 4-hydroxybenzoic acid, 3,4-dihydroxybenzaldehyde, phloroglucinaldehyde, protocatechuic acid, 3,5-dihydroxybenzoic acid, methyl-3,5-dihydroxybenzoate, methyl-3,4-dihydroxybenzoate, 4-methoxysalicilic acid, ferulic acid, isoferulic acid, ferulic acid sulfate were obtained from Sigma-Aldrich; isoferulic acid-3-O-sulfate and ferulic acid-4-O-glucuronide were obtained from Toronto Research Chemicals Inc. All solvents were HPLC grade and were obtained from Sigma-Aldrich or Fisher Scientific.

*Solid phase extraction*

Anthocyanins and phenolic acids were extracted from urine using a validated method [4] with minor modifications. Briefly, 1 mL of urine was spiked with an internal standard (i.e. 3,5-dichloro-4-hydroxybenzoic acid) and subsequently extracted using solid phase extraction cartridges (Strata-X 33μm polymeric sorbent 500 mg/6m; Phenomenex). These were washed with two column volumes of 1% formic acid in water, dried for 30 min under vacuum, soaked in 1% formic acid in methanol for 10 min and then eluted into glass vials with 7 mL of 1% formic acid in methanol. Samples were evaporated to complete dryness under Speedvac centrifugal evaporator at room temperature. The dried samples were resuspended in 250 μL of 1% formic acid in water by 30 s vortexing and 15 min ultrasound sonicating. Samples were stored at -80 °C until analysis.

*UPLC-MS/MS analysis*

The UPLC-electrospray ionisation- MS/MS system consisted of an Aquity UPLC Hclass (Waters) coupled to a Xevo TQ-S micro electrospray ionisation mass spectrometer (Waters) operated using MassLynx software (V4.1, Waters Inc, USA). Compound separation was achieved using an Aquity UPLC HSS T3 1.8µm column (2.1 x 100mm) attached to a Van guard pre-column of the same material and pore size, maintained at 45°C with a flow of 0.65 mL/min and a sample injection volume of 2 μL. The mobile phase consisted of 0.1/99.9 v/v formic acid/water (A) and 0.1/99.9 v/v formic acid/acetonitrile (B); and a mobile phase gradient consisting of: 1% B at 0 min, 1% B at 1 min, 30 % B at 10 min, 95 % B at 12 min, 95% B at 13 min, 99% B at 13.10 min, 99% B at 16 min. A scheduled multiple reaction monitoring (sMRM) method was developed for a total of 24 standard compounds (**Supplementary Table 1**), using sMRM transitions, and optimal sMRM modes (i.e. negative or positive) and collision energies determined by syringe infusion of analytical standards. Isoferulic acid-3-O-sulfate and ferulic acid-4-O-glucuronide were infused for MRM optimization, and standard calibration curves were run. Additionally, putative glucuronide and sulfate conjugated phenolic acid metabolites were added to the sMRM method, even though analytical standards were not commercially available. Retention times and sMRM transitions were tentatively identified by injecting a pooled extract of urine (i.e. using urine collected after blackcurrant drink intake from n=3 participants and during all 6 time intervals per each visit). sMRM transitions were taken from the literature [4-5] or derived from the fragmentation pattern of the phenolic acid aglycones by adding the m/z of glucuronide or sulfate to the precursor ion and including the appropriate MS/MS fragment.

Quantification was established using the most intense sMRM transition and standard curves of analytical standards. Where pure standards were not available, quantification was conducted relative to standard curves of compounds with similar structure. The limit of detection was established for each compound as the concentration of a peak with a signal to noise ratio of 3. From all 52 compounds originally considered in the preliminary analysis by UPLC-MS/MS, 15 were identified as phenolic compounds derived from blackcurrant drink in the plasma, and 30 in urines.

**Supplementary Table 1.** Scheduled multiple reaction monitoring **(**sMRM) transitions, parameters and detection limits of 29 identified blackcurrant drink polyphenols.

*LOD, limit of detection; MW, molecular weight; NO STD, quantification was conducted relative to standard curves of compounds with similar structure as pure standards was not available; sMRM, scheduled multiple reaction monitoring, V, Volts*

| **Metabolite** | **ION MODE** | **MW** | **sMRM ion transitions (m/z)** | **Collision energy (V)** | **LOD (nM)** | **R^2^** |
| --- | --- | --- | --- | --- | --- | --- |
| ***Anthocyanins*** |  |  |  |  |  |  |
| Cyanidin-3-glucoside | + | 449.4 | 287 | 20 | 0,02 | 1 |
| Delphinidin-3-glucoside | + | 465.3 | 303 | 20 | 0,05 | 1 |
| Cyanidin-3-rutinoside | + | 630.9 | 287 | 17 | 0,02 | 1 |
| Delphinidin-3-rutinoside | + | 611.5 | 303 | 20 | 5,4 | 1 |
| ***Aglycones*** |  |  |  |  |  |  |
| Cyanidin aglycone | + | 287 | 137 | 20 | 2.7 | NO STD |
| Vanillic acid | + | 168.2 | 93 | 12 | 11,5 | 0.998 |
| Isovanillic acid | + | 168.2 | 93 | 12 | 2,3 | 0.995 |
| Syringic acid | + | 198.2 | 140 | 15 | 15,8 | 0.999 |
| 4-hydroxybenzaldehyde | - | 122.1 | 92 | 20 | 4,6 | 0.996 |
| Hippuric acid | - | 179.2 | 134 | 11 | 12,9 | 0.990 |
| 2-hydroxybenzoic acid | - | 138.1 | 93 | 10 | 3,8 | 0.992 |
| 3-hydroxybenzoic acid | - | 138.1 | 93 | 10 | 16,7 | 0.989 |
| 4-hydroxybenzoic acid | - | 138.1 | 93 | 13 | 19,4 | 0.992 |
| 3,4-dihydroxybenzaldehyde | - | 138.1 | 108 | 15 | 0,03 | 0.990 |
| Phloroglucinaldehyde | - | 154.1 | 151 | 12 | 0,06 | 0.988 |
| Protocatechuic acid | - | 154.1 | 81 | 20 | 0,30 | 0.999 |
| 3,5-dihydroxybenzoic acid | - | 154.1 | 67 | 15 | 2,7 | 0.998 |
| Methyl 3,5 -dihydroxybenzoate | - | 168.1 | 108 | 15 | 9,1 | 0.998 |
| Ferulic acid | - | 194.2 | 178 | 10 | 0,1 | 1 |
| Isoferulic acid | - | 194.2 | 178 | 10 | 0,2 | 1 |
| ***Glucuronides*** |  |  |  |  |  |  |
| Ferulic acid glucuronide | - | 370.3 | 193 | 19 | 0,04 | 1 |
| Isoferulic acid glucuronide | - | 370.3 | 193 | 19 | 16 |  |
| Cyanidin glucuronide | + | 463 | 287 | 10 | 0.2 | NO STD |
| Cyanidin methyl glucuronide | + | 477 | 301 | 20 | 1.2 | NO STD |
| Delphinidin methyl glucuronide | + | 491 | 317 | 15 | 0.05 | NO STD |
| Isovanillic acid glucuronide | - | 344.3 | 167 | 15 | 0.02 | NO STD |
| ***Sulfates*** |  |  |  |  |  |  |
| Ferulic acid sulfate | - | 274.2 | 178 | 20 | 4.6 | NO STD |
| Isoferulic acid sulfate | - | 274.2 | 178 | 20 | 0,02 | 1 |
| Benzoic acid-sulfate | - | 217 | 173 | 15 | 0.2 | NO STD |

**References:**

1. Wu X, Gu L, Prior RL, McKay S. Characterization of anthocyanins and proanthocyanidins in some cultivars of Ribes, Aronia, and Sambucus and their antioxidant capacity. J Agric Food Chem. 2004;52(26):7846-7856.

2. Hollands WJ, Voorspoels S, Jacobs G, et al. Development, validation and evaluation of an analytical method for the determination of monomeric and oligomeric procyanidins in apple extracts. J Chromatogr A. 2017;1495:46-56.

3. Hollands WJ, Tapp H, Defernez M, et al. Lack of acute or chronic effects of epicatechin-rich and procyanidin-rich apple extracts on blood pressure and cardiometabolic biomarkers in adults with moderately elevated blood pressure: a randomized, placebo-controlled crossover trial. Am J Clin Nutr. 2018;108(5):1006-1014.

4. de Ferrars RM, Czank C, Saha S, et al. Methods for isolating, identifying, and quantifying anthocyanin metabolites in clinical samples. Anal Chem. 2014;86(20):10052-10058.

5. Kalt W, Liu Y, McDonald JE, Vinqvist-Tymchuk MR, Fillmore SA. Anthocyanin metabolites are abundant and persistent in human urine. J Agric Food Chem. 2014;62(18):3926-3934.

# **Supplementary Figures**

**Supplementary Figure 1**


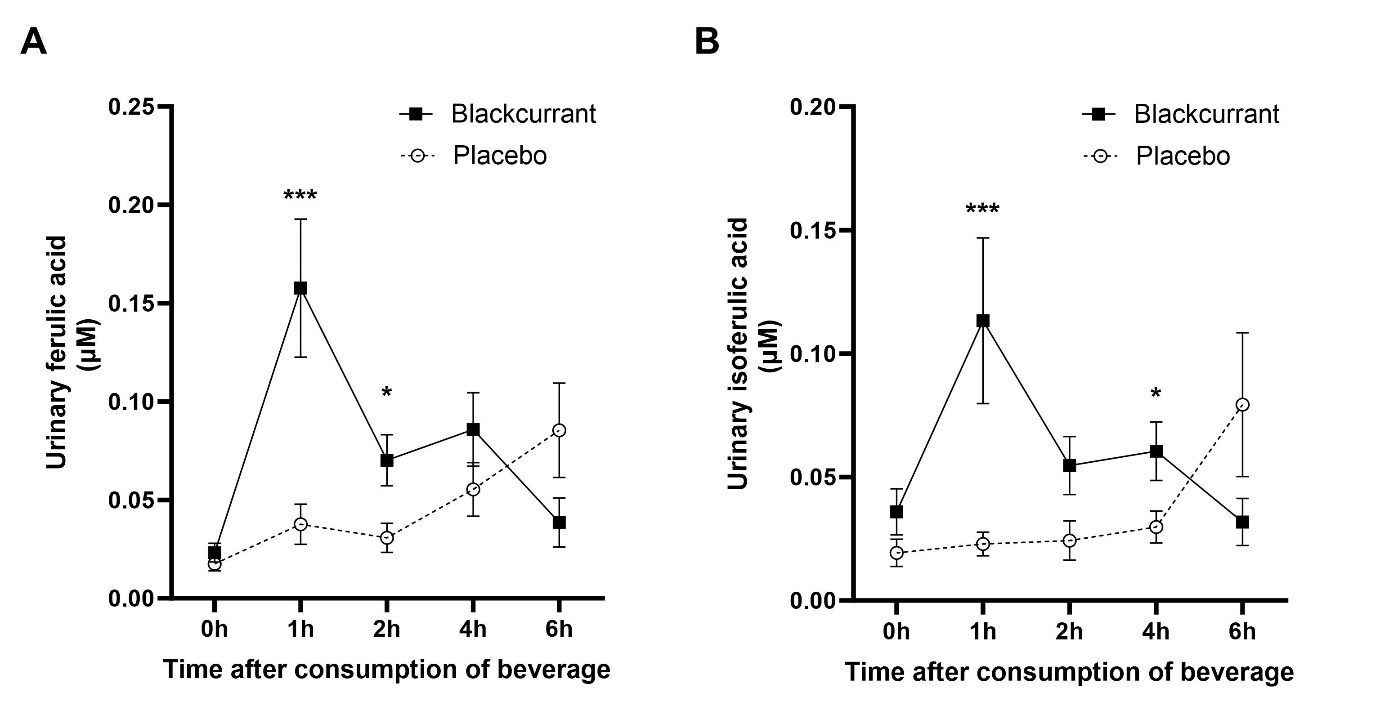


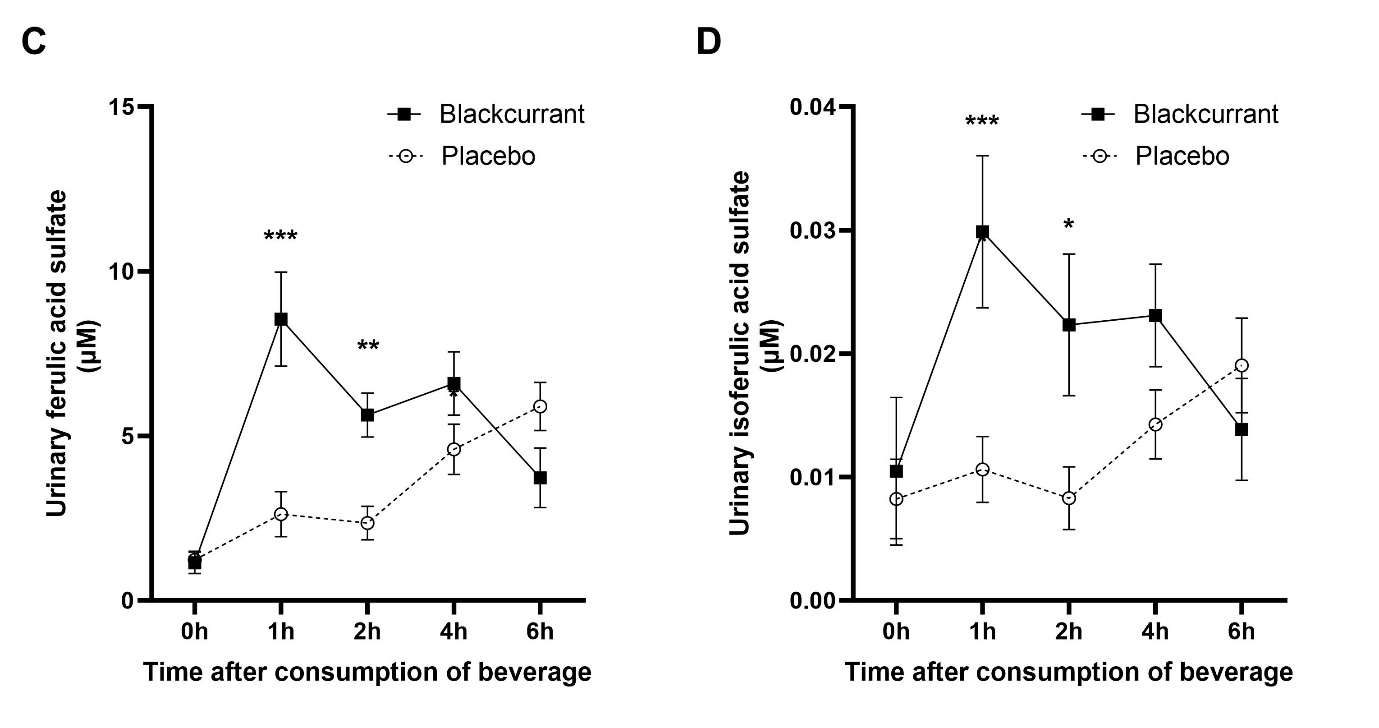


**
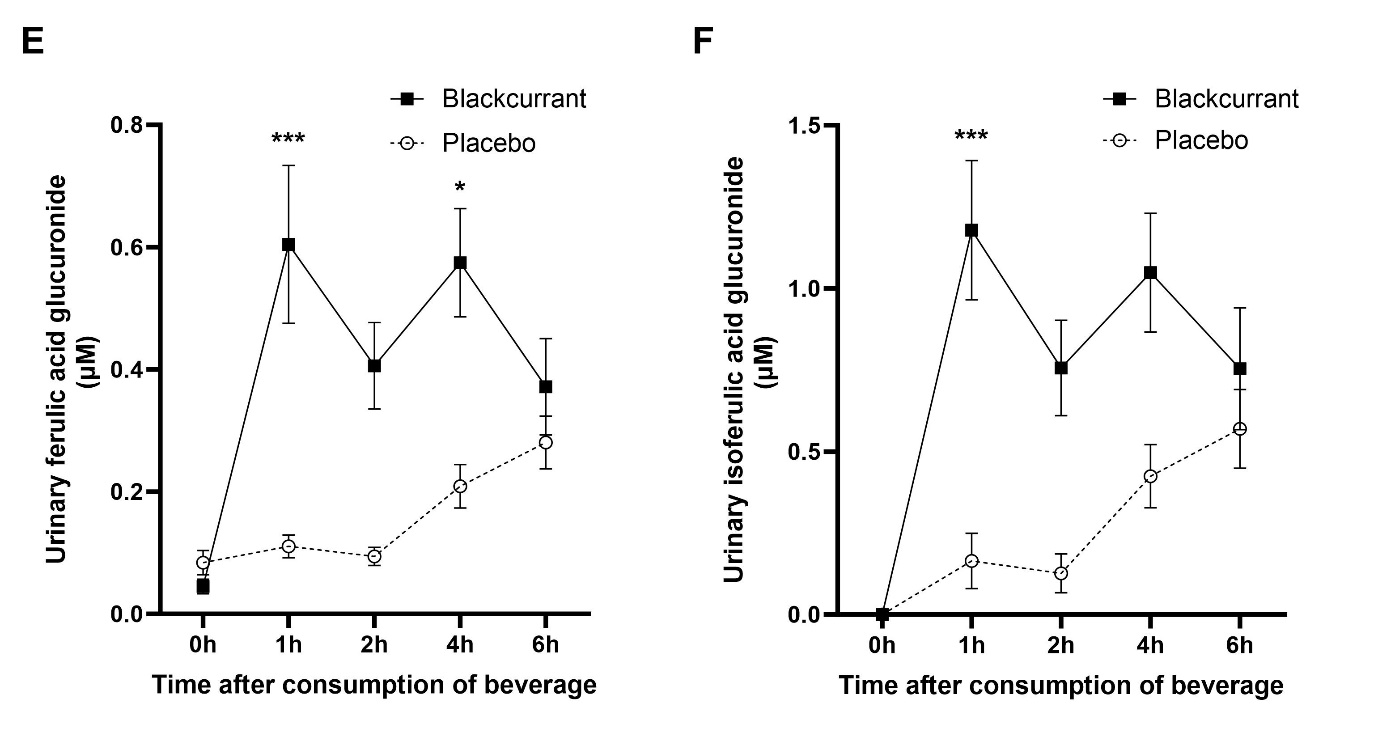
**

**
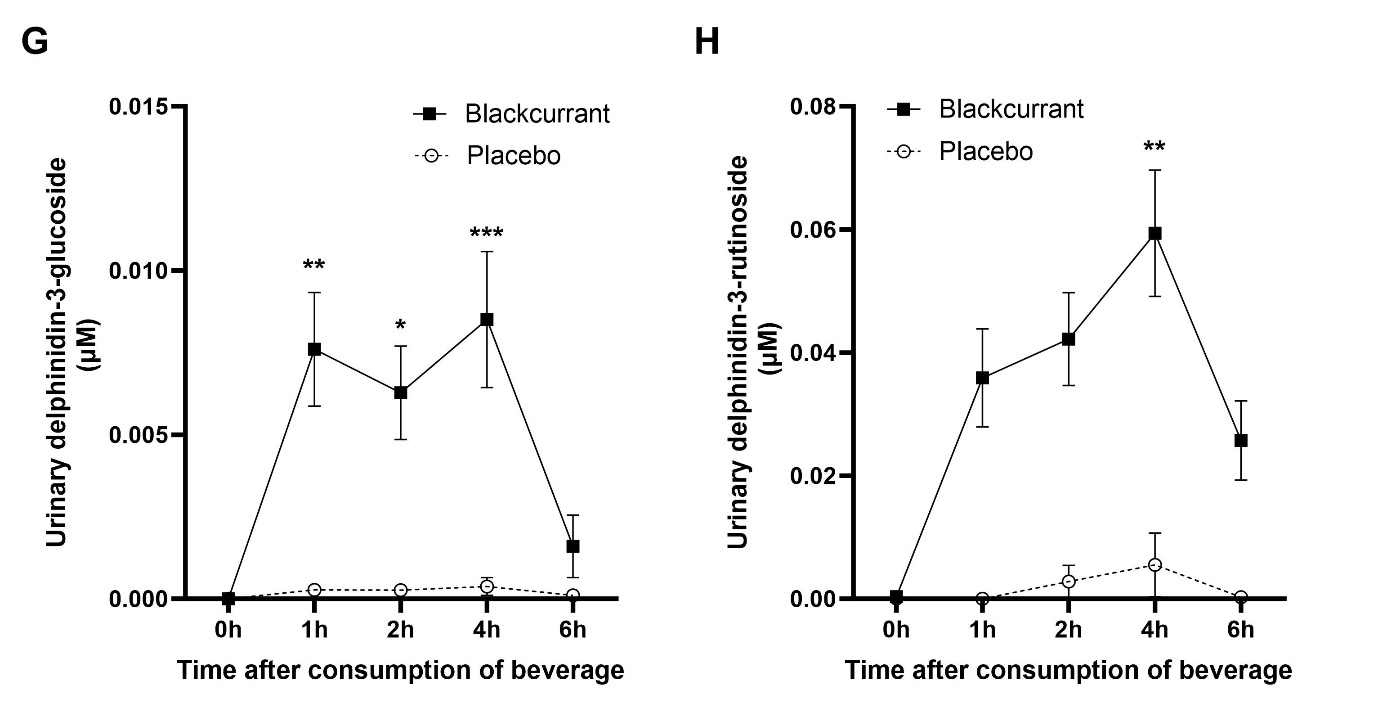
**

**
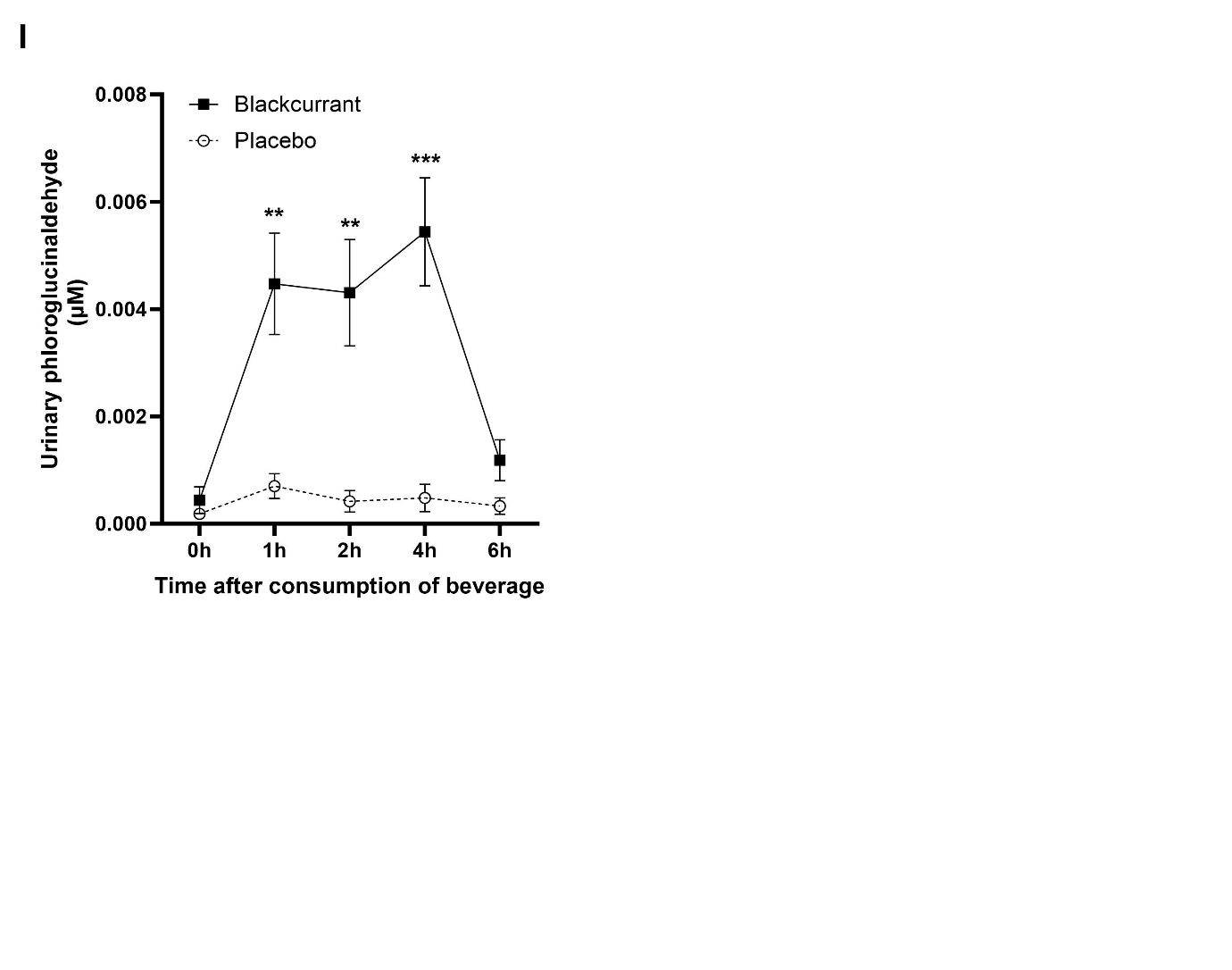
**

**Supplementary Figure 1: Changes in urinary excretions of parent anthocyanins,** **anthocyanin conjugates and phenolic metabolites during 0-6h after consumption of the blackcurrant beverage or placebo together with a high-fat breakfast.**

Data are mean ± SEM (n=12). Data were analyzed by using a linear mixed model with treatment (2 treatments: blackcurrant compared to placebo beverage) and time (4 levels) as factors, and post hoc analyses were conducted by using a Bonferroni multiple-comparisons test. In response to blackcurrant beverage compared to placebo, urinary excretions of **A**: ferulic acid during 0-1 and 1-2h, and **B**: isoferulic acid during 0-1 and 2-4h post-consumption were significantly increased; urinary excretions of **C**: ferulic acid sulfate and **D:** isoferulic acid sulfate during 0-1 and 1-2h post-consumption were significantly increased; urinary excretions of **E:** ferulic acid glucuronide during 0-1 and 2-4h and **F:** isoferulic acid glucuronide during 0-1h post-consumption were significantly increased; urinary excretions of **G:** delphinidin-3-glucoside during 0-1, 1-2h, and 2-4h, and **H:** delphinidin-3-rutinoside during 2-4h post-consumption were significantly increased; urinary excretions of **I:** phloroglucinaldehyde during 0-1, 1-2h, and 2-4h were significantly increased. *,**,*** Significantly different from the control drink at the specified time point: **P* < 0.05, ***P* < 0.01, ****P* < 0.001.

**Supplementary Figure 2**


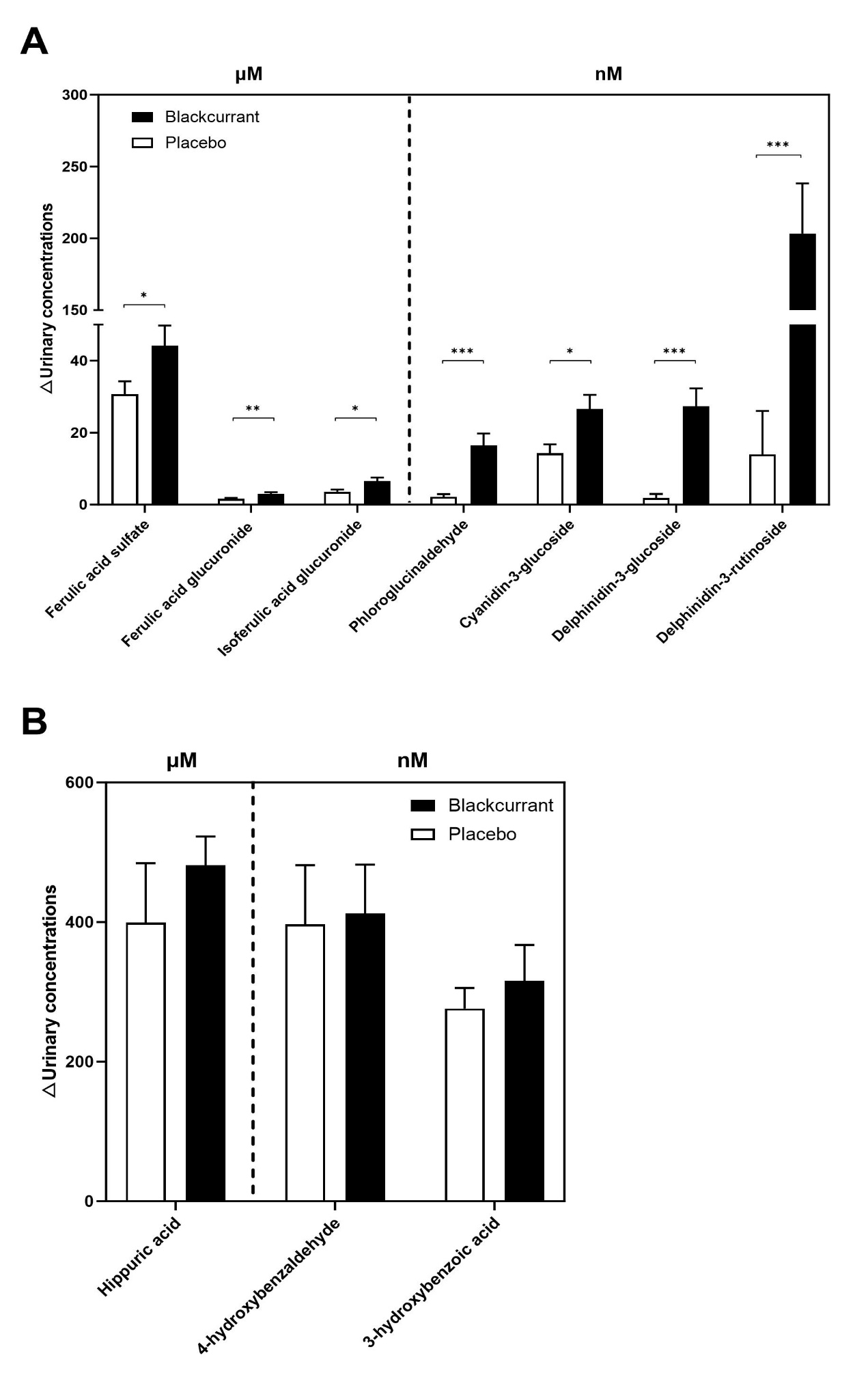


**Supplementary Figure 2: Changes from baseline in urinary excretions of parent anthocyanins, anthocyanin conjugates and phenolic metabolites over the 24h period after consumption of the blackcurrant beverage or placebo together with a high-fat breakfast.**

Data are mean ± SEM (n=12). Values of metabolites compounds are expressed as either μM or nM as shown. Differences from baseline were analyzed by using a linear mixed model with treatment (2 treatments: blackcurrant compared to placebo beverage) and time (4 levels) as factors, and post hoc analyses were conducted by using a Bonferroni multiple-comparisons test. **A:** Urinary excretions of ferulic acid sulfate, ferulic acid glucuronide, isoferulic acid glucuronide, phloroglucinaldehyde, cyanidin-3-glucoside, delphinidin-3-glucoside and delphinidin-3-rutinoside were significantly increased over the whole 24 h period following consumption of the blackcurrant beverage compared to the placebo. **B:** there are no significant increases in urinary excretions of hippuric acid, 4-hydroxybenzaldehyde and 3-hydroxybenzoic acid over the whole 24 h period following consumption of the blackcurrant beverage compared to the placebo. *,**,*** Significantly different from the control drink at the specified time point: **P* < 0.05, ***P* < 0.01, ****P* < 0.001.

**Supplementary Figure 3**

**
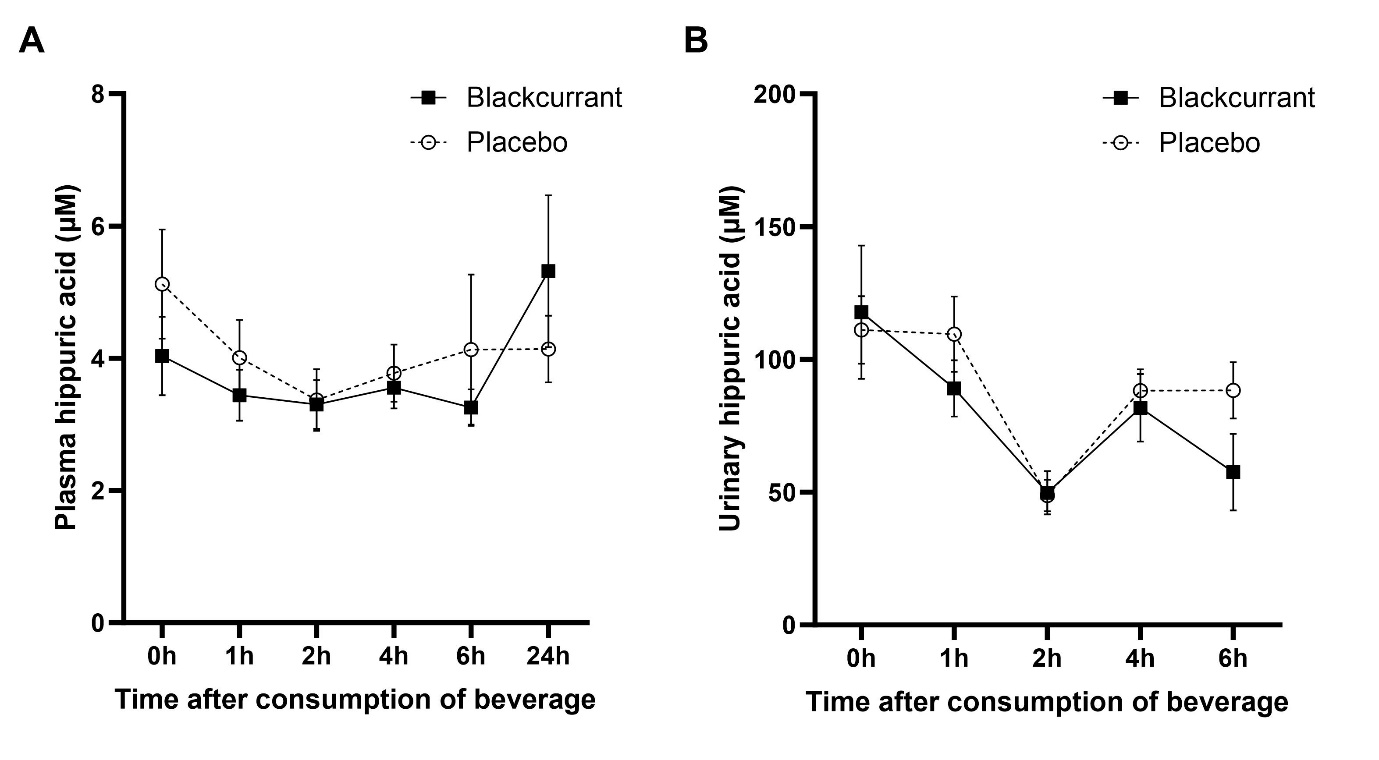
**

**
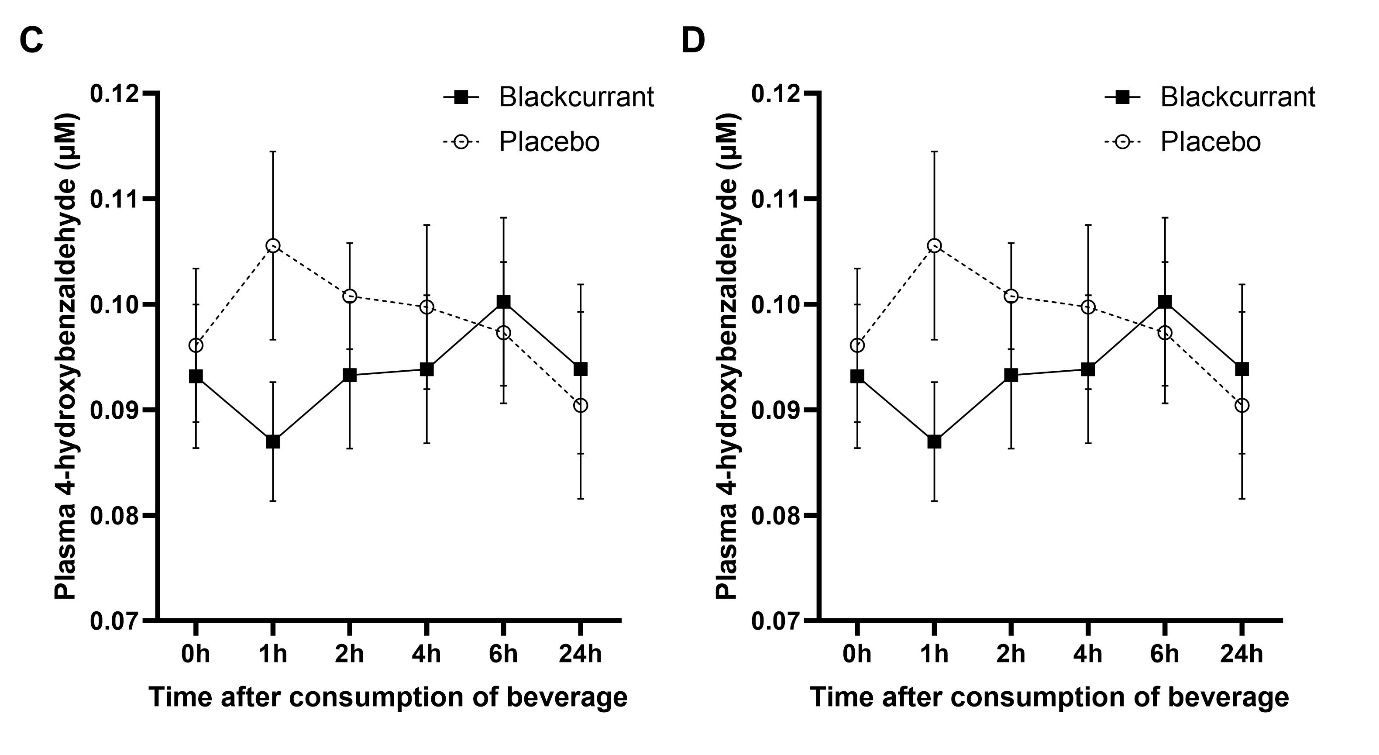
**

**
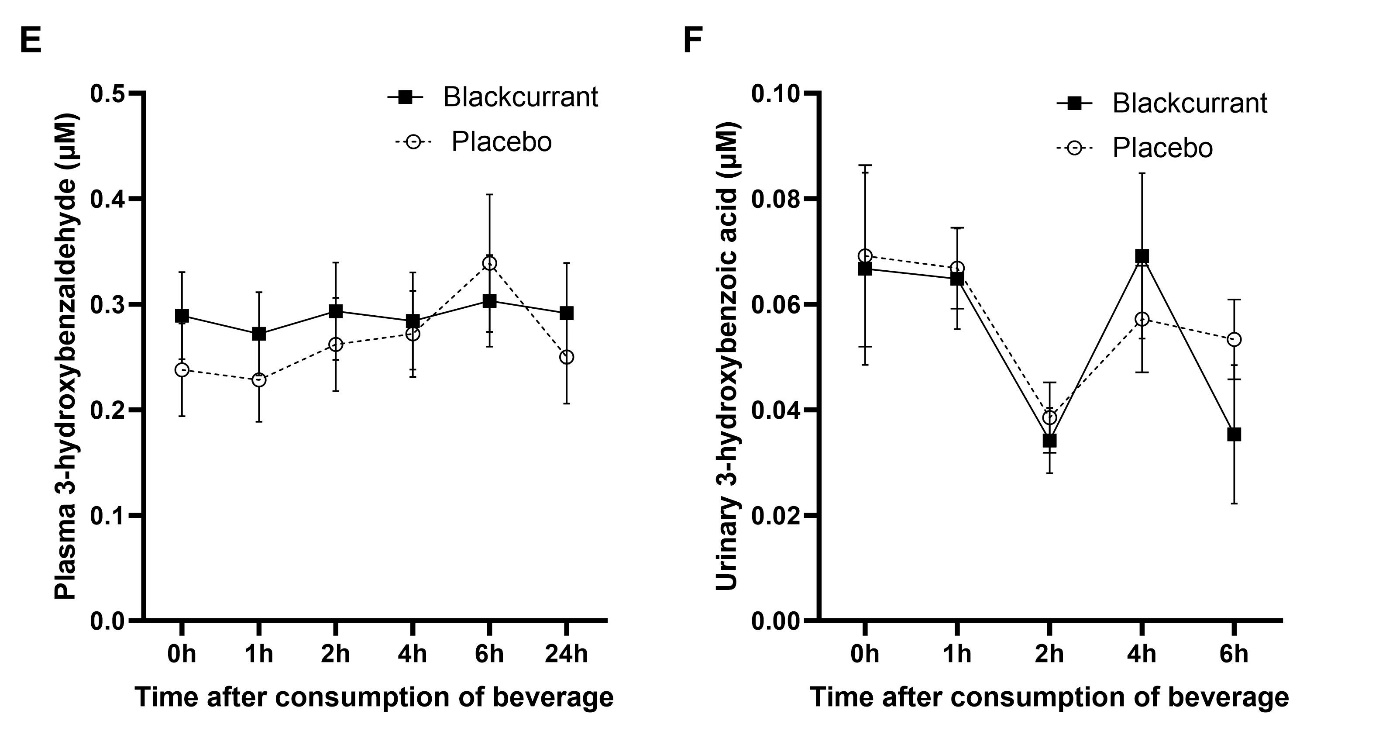
**

**Supplementary Figure 3: Changes in plasma and urinary concentrations of phenolic metabolites associated with FMD, SBP and IL-8 after consumption of the blackcurrant beverage or placebo together with a high-fat breakfast.**

Data are mean ± SEM (plasma: n= 21; urine: n=12). Data were analyzed by using a linear mixed model with treatment (2 treatments: blackcurrant compared to placebo beverage) and time (4 levels) as factors, and post hoc analyses were conducted by using a Bonferroni multiple-comparisons test. Plasma concentrations of hippuric acid is associated with FMD, but there are no significant increase in either **A:** plasma concentration or **B:** urinary excretions of hippuric acid after the consumption of the blackcurrant beverage compared to placebo. Plasma concentrations of 4-hydroxybenzaldehyde is associated with SBP, but there are no significant increase in either **C:** plasma concentration or **D:** urinary excretions of 4-hydroxybenzaldehyde after the consumption of the blackcurrant beverage compared to placebo. Plasma concentrations of 3-hydroxybenzoic acid is associated with IL-8, but there are no significant increase in either **E:** plasma concentration or **F:** urinary excretions of 3-hydroxybenzoic acid after the consumption of the blackcurrant beverage compared to placebo.
